# Two New Cytotoxic Compounds from a Deep-Sea *Penicillum citreonigrum* XT20-134

**DOI:** 10.3390/md17090509

**Published:** 2019-08-29

**Authors:** Xi-Xiang Tang, Shun-Zhi Liu, Xia Yan, Bo-Wen Tang, Mei-Juan Fang, Xiu-Min Wang, Zhen Wu, Ying-Kun Qiu

**Affiliations:** 1 Key Laboratory of Marine Biogenetic Resources, Third Institute of Oceanography State Oceanic Administration, Xiamen 361005, China; 2 Fujian Provincial Key Laboratory of Innovative Drug Target Research, School of Pharmaceutical Sciences, Xiamen University, South Xiang-An Road, Xiamen 361102, China; 3 Li Dak Sum Yip Yio Chin Kenneth Li Marine Biopharmaceutical Research Center, Ningbo University, Ningbo 315832, China

**Keywords:** *Penicillum citreonigrum* XT20-134, MCCC 3A00956, deep-sea fungus, cytotoxic activity

## Abstract

*Penicillum citreonigrum* XT20-134 (MCCC 3A00956) is a fungus with cytotoxic activity, derived from deep-sea sediment. Five new compounds, adeninylpyrenocine (**1**), 2-hydroxyl-3-pyrenocine-thio propanoic acid (**2**), ozazino-*cyclo*-(2,3-dihydroxyl-trp-tyr) (**3**), 5,5-dichloro-1-(3,5-dimethoxyphenyl)-1,4-dihydroxypentan-2-one (**4**), and 2,3,4-trihydroxybutyl cinnamate (**5**), together with 19 known compounds (**6**–**24**), were isolated from an ethyl acetate (EtOAc) extract of its fermentation. The structures of the new compounds were comprehensively characterized by high-resolution electrospray ionization-mass spectrometry (HR-ESI-MS), 1D and 2D nuclear magnetic resonance (NMR). All isolates were evaluated for their cytotoxic activities. The heteroatom-containing new compounds **2** and **4** showed potent cytotoxicity to the human hepatoma tumor cell Bel7402 with IC_50_ values of 7.63 ± 1.46, 13.14 ± 1.41 μM and the human fibrosarcoma tumor cell HT1080 with IC_50_ values of 10.22 ± 1.32, 16.53 ± 1.67 μM, respectively.

## 1. Introduction

Considering the extreme environment of high salinity, darkness, high pressure, and high/low temperature [1], the discovery of new lead compounds from deep-sea microorganisms has become a hot topic in natural products research. Deep-sea fungi are attracting great interest because of their relatively large genome size, which may produce more second metabolites compared with bacteria. Cancer is the leading lethal disease in the world and deep-sea microorganism-originated compounds are thought to be the new anti-tumor drugs repository [2,3]. Recently, the new antitumor compounds diketopiperazine, cytochalasan alkaloids, chromone-derived polyketides, etc., have been isolated from deep-sea fungi [4,5,6].

**Figure 1:**
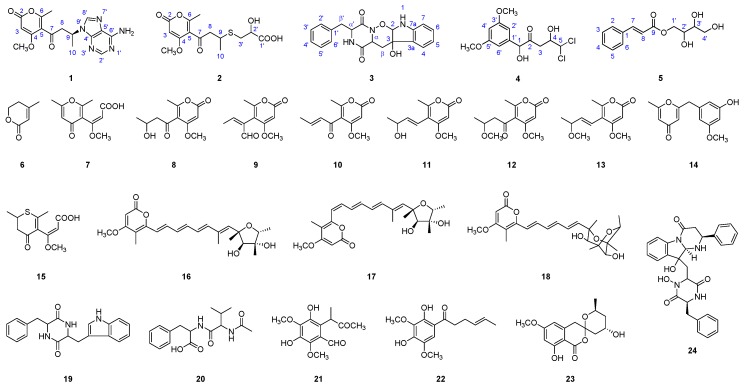
Structures of compounds **1**–**24** isolated from an extract of *Penicillum citreonigrum* XT20-134.

During the previous studies, several new cytotoxic compounds were characterized from deep-sea microbial resources [7,8,9,10,11]. In the present study, *Penicillum citreonigrum* XT20-134 (MCCC 3A00956), a fungal strain that originated from the deep-sea sediment in the southeast Indian Ocean, was found to possess cytotoxic activity. From its ethyl acetate (EtOAc) extract, five new compounds, adeninylpyrenocine (**1**), 2-hydroxyl-3-pyrenocine-thio propanoic acid (**2**), ozazino-*cyclo*-(2,3-dihydroxyl-*trp*-*tyr*) (**3**), 5,5-dichloro-1-(3,5-dimethoxyphenyl)-1,4-dihydroxypentan-2-one (**4**), and 2,3,4-trihydroxybutyl cinnamate (**5**) were isolated together with 19 known compounds (**6**–**24**) (Figure 1). The structures of the new compounds were comprehensively characterized by high-resolution electrospray ionization-mass spectrometry (HR-ESI-MS), 1D and 2D nuclear magnetic resonance (NMR). Their cytotoxic activities were studied. The new heteroatom-containing compounds **2** and **4** showed potent cytotoxicity to the human hepatoma tumor cell Bel7402 with IC_50_ values of 7.63 ± 1.46 and 13.14 ± 1.41 μM, and the human fibrosarcoma tumor cell HT1080 with IC_50_ values of 10.22 ± 1.32 and 16.53 ± 1.67 μM, respectively.

## 2. Results

### 2.1. Structural Identification of New Compounds

**Figure 2:**
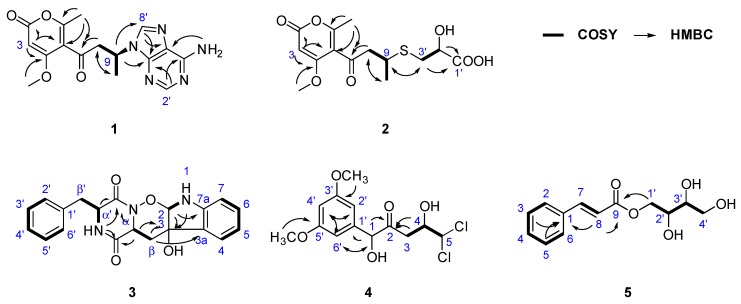
Key ^1^H–^1^H COSY, HMBC correlations of compounds **1**–**5**.

**Figure 3:**
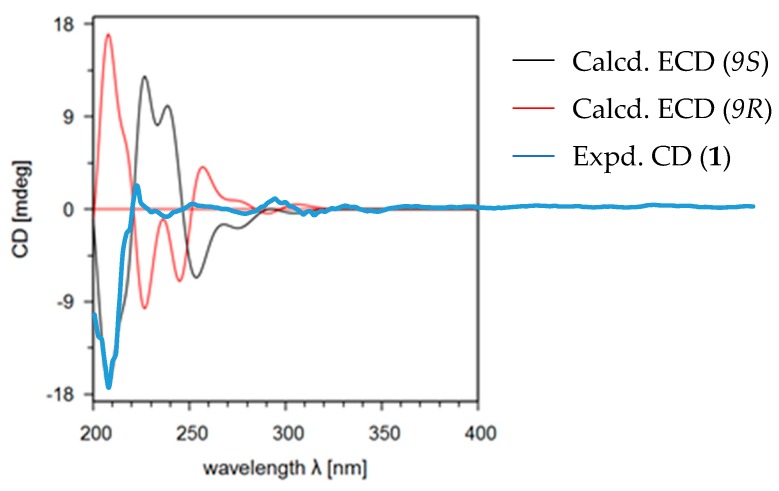
Calculated (*9R* and *9S*) and experimental electronic circular dichroism (ECD) spectra of compound **1**.

Adeninylpyrenocine (**1**) was isolated as a white amorphous powder. The infrared (IR) spectrum of **1** indicated the presence of conjugated ketone and conjugated lactone carbonyl signals at 1695 and 1642 cm^−1^, respectively. Its molecular formula of C_16_H_17_N_5_O_4_ was established by HR-ESI-MS at 344.1357 [M + H]^+^ (calcd. for 344.1353, C_16_H_18_N_5_O_4_). The unsaturation degree of 11 indicated the presence of heteroaromatic rings. With the aid of heteronuclear single quantum coherence (HSQC) spectra, three singlet signals in the low field of ^1^H NMR at *δ_H_* 8.21 (1H, s, H-8’), 8.11 (1H, s, H-2’), and 5.62 (1H, s, H-3) were attributed to olefinic protons. Their corresponding carbon signals were found in ^13^C NMR at *δ_C_* 140.3, 152.6, and 87.8, respectively. The signal at *δ_H_* 7.16 (2H, br.s, 6’-NH_2_) should be assigned to the primary amino group due to the absence of carbon correlation in the HSQC spectrum. In the *sp*^3^ region of the ^1^H NMR, two singlet signals, belonging to a methoxyl at *δ_H_* 3.74 (3H, s, 4-OCH_3_) and a methyl at *δ_H_* 1.85 (3H, s, 6-CH_3_), could be found. In addition, a CH_2_–CH–CH_3_ fragment could be established, based on the ABXC_3_ spin system at *δ_H_* [3.78 (1H dd, *J* = 17.6, 7.9 Hz) and 3.35 (1H dd, *J* = 17.6, 6.0 Hz), H-8], *δ_H_* 5.06 (1H, m, H-9), and the doublet methyl signal and 1.53 (3H, d, *J* = 6.0 Hz, H-10). Considering the five nitrogen atoms in the molecular formula and the carbon signals at *δ_C_* 156.4, 152.6, 149.5, 140.3, and 119.5, an adenine moiety is present in **1**. With the help of distortionless enhancement by polarization transfer (DEPT-135) along ^13^C NMR, the four quaternary carbons at *δ_C_* 156.4, 152.6, 149.5, 140.3, and the methine at *δ_C_* 87.8 were attributed to an α-pyrone structure unit. In the ^1^H–^1^H homonuclear chemical shift correlation spectroscopy (COSY) spectrum, only the correlations in the CH_2_CH–CH_3_ were presented. In the ^1^H detected heteronuclear multiple bond correlation (HMBC) spectrum, key correlations were found to reveal the total structure. The correlations between H-9 (*δ_H_* 5.06) and C-4’ (*δ_C_* 149.5) and C-8’ (*δ_C_* 140.3) indicated that the adenine moiety was connected to C-9 at N-9’. The conjugated ketone signal at *δ_C_* 198.8 (C-7) in ^13^C NMR, is considered to link to C-8, due to the HMBC correlation between H-8 and C-7. The other two methyl groups were also assigned, as shown in Figure 2. The theoretical electronic circular dichroism (ECD) spectra of *9R*-**1** and *9S*-**1** were further calculated and compared with the experimental ones to determine the absolute configurations. As shown in Figure 3, the experimental ECD spectrum was similar to the calculated ECD spectrum of *9S*-**1** and the absolute configuration of **1** was determined as *9S*.

We isolated 2-hydroxyl-3-pyrenocine-thio propanoic acid (**2**, a pair of epimers with a ratio of 1:2) as a light yellow powder. In the IR spectrum of **2**, unconjugated carbonyl and α-pyrone ketone signals emerged at 1714 and 1626 cm^−1^, respectively. Its molecular formula of C_14_H_18_O_7_S, which gave six degrees of unsaturation, was established by the positive HR-ESI-MS ion peaks at *m*/*z* 331.0845 [M + H]^+^, 353.0670 [M + Na]^+^ and negative ion peaks at *m*/*z* 329.0706 [M − H]^−^, respectively. The presence of a sulfur atom was supported by the isotope quasi-molecular ion peaks at *m*/*z* 333.0808 [M(^34^S) + H]^+^, 355.0627 [M(^34^S) + Na]^+^ and 331.0662 [M(^34^S) – H]^−^, respectively. In the ^1^H NMR spectrum of **2**, an α-pyrone olefinic proton, a methyl and a methoxyl signal were found at *δ_H_* 5.69 (1H, s, H-3), 1.23 (1H, d, *J* = 6.8 Hz, H-10) and 3.87 (3H, s, 4-OCH_3_), respectively. In the ^1^H–^1^H COSY spectrum, the correlations between *δ_H_* 5.69 (H-10), *δ_H_* 3.30 (H-9) and *δ_H_* 2.99 & 2.90 (H-8) revealed the presence of a CH_2_CH–CH_3_ structure unit. In the ^13^C NMR, signals belong to the pyrenocine moiety were similar to those in **1**, except for the C-9 signal at *δ_C_* 36.3, indicating that they differed in the substituent at C-9 (Table 1). The adeninyl signals were absent in the NMR data of **2**. Except for the adeninyl signals, the ^1^H-NMR of **2** presented other ABX system signals at *δ_H_* 4.09 (1H, m, H-2’) and *δ_H_* [2.83 (1H, dd, *J* = 13.5, 5.1 Hz) and 2.72 (1H, dd, *J* = 13.5, 9.5 Hz), H-3’]; and three additional carbon signals were at *δ_C_* 174.5 (C-1’), 71.1 (C-2’) and 34.7 (C-3’). With the help of ^1^H–^1^H COSY and HMBC spectra, they were attributed to a –CH_2_–CH(OH)–COOH structure fragment. The HMBC correlations between H-3’ (*δ_H_* 2.83, 2.71) and C-9 (*δ_C_* 36.3), and between H-9 (*δ_H_* 3.30) and C-3’ (*δ_C_* 34.7) revealed that the two structure units connected at C-9 and C-3’. The ^1^H-NMR and ^13^C-NMR signals at positions C-1’, 2’, 3’ and C-8, 9, whose peak intensities were halved and appeared in pairs, indicated that compound **2** is a pair of epimers (Figure 2). Considering the similar structure unit in **1**, the absolute configuration of C-9 is *S*, while C-2’ contains the pair of *R* and *S* configurations.

Ozazino-*cyclo*-(2,3-dihydroxyl-*trp*-*tyr*) (**3**) was isolated as a white powder. The HR-ESI-MS cationized ion peaks revealed the presence of three nitrogen atoms. In the IR spectrum of **3**, amide carbonyl signal was found at 1669 cm^−1^. The ultravoilet (UV) maximum absorption wavelengths at *λ*_max_ (log ε) 240 (1.92) nm and 299 (1.55) nm, belong to the amide carbonyl and aromatic rings, respectively. In the ^1^H NMR spectrum of **3**, a set of *mono*-substituted benzene ring signals could be found at *δ_H_* 6.98 (2H, br. d, *J* = 7.2 Hz, H-2’, 6’), 6.86 (2H, br. t, *J* = 7.4 Hz, H-3’, 5’) and 6.75 (1H, m, H-4’), respectively. Another *ortho*-substituted benzene ring was elucidated by the signals at *δ_H_* 6.90 (1H, br. d, *J* = 7.2 Hz, H-4), 6.56 (1H, br. t, *J* = 7.0 Hz, H-5), 7.00 (1H, br. t, *J* = 7.5 Hz, H-6), and 6.56 (1H, br. d, *J* = 7.7 Hz, H-7). In addition, two ABX spin system signals were present at *δ_H_* 4.19 (1H, dd, *J* = 5.0, 2.8 Hz, H-α′), [2.87 (1H, dd, *J* = 13.9, 2.8 Hz) and 2.74 (1H, dd, *J* = 13.8, 5.0 Hz), H-β′], and at *δ_H_* 4.17 (1H, dd, *J* = 9.1, 4.8 Hz, H-α), [1.91 (1H, dd, *J* = 13.6, 4.8 Hz) and 1.03 (1H, dd, *J* = 13.5, 9.1 Hz) H-β], and were attributed to two –CH–CH_2_– structure units. All these fragments were confirmed by the ^13^C NMR and DEPT spectra. In addition, two amide carbonyls at *δ_C_* 166.1 and 161.5, together with a quaternary carbon at *δ_C_* 74.7 and a methylidyne *δ_C_* 99.3, indicated that **3** is a compound comprising two amino acid units, one of which is phenylalanine and the other one is a tryptophane-like structure unit. Similar structures have been isolated from *P. citroviride* [12] and from *Penicillium* sp. [13]. In the HSQC spectrum of **3**, the proton signals at *δ_H_* 7.96 (α′-NH) and *δ_H_* 6.57 (1-NH) were attributed to two exchangeable protons, for the absence of correlation with carbon. With the help of the ^1^H–^1^H COSY spectrum, the signals belonging to the phenylalanine unit were attributed (Figure 2). The COSY correlation between *δ_H_* 6.57 (1-NH) and *δ_H_* 5.10 (1H, d, *J* = 2.8 Hz, H-2), together with the HMBC correlation between H-2 and C-3 (*δ_C_* 74.6), revealed that C-2 and C-3 of the tryptophane unit were oxygen-connected. The HMBC correlations between *δ_H_* 7.96 (α′-NH), 4.17 (H-α), 4.19 (H-α’), and *δ_C_* 166.1 (C=O), 161.5 (C=O’) indicated the *cyclo*-dipeptide structure. Comparing the NMR data of **3** with those of *cyclo*-(L-tryptophyl-L-phenylalanyl) (**19**), a known compound reported previously [14], the chemical shift of 1-NH was significantly deduced from *δ_H_* 10.83 to *δ_H_* 6.57, indicating that the double bond between C-2 and C-3 in the indole ring disappeared. Moreover, absence of the α-NH signal (*δ_H_* 7.96 in **19**), and the high-field shifting of C=O’ (*δ_C_* 167.3 in **19** and *δ_C_* 161.5 in **3**) were observed. Considering that the degree of unsaturation in **3** is 13, a six-membered ring between α-N and 2-O, connected by an ozazino bond, is prefered. Thus, the structrue of 3 is elucidated as ozazino-*cyclo*-(2,3-dihydroxyl-trp-tyr).

We isolated 5,5-dichloro-1-(3,5-dimethoxyphenyl)-1,4-dihydroxypentan-2-one (**4**) as a white powder. The HR-ESI-MS cationized ion peaks indicated a molecular formula of C_13_H_16_Cl_2_O_5_. The presence of the two chlorine atoms was confirmed by the isotope ion peak relative high ratio of 9:6:1. In the IR spectrum of **4**, an unconjuated ketone carbonyl signal was at 1714 cm^−1^. A symmetrical 1,3,5-trisubstituted benzene ring was deduced by the ^1^H NMR signal at *δ_H_* 6.37 (2H, d, *J* = 2.2 Hz, H-2’, 6’) and *δ_H_* 6.27 (1H, d, *J* = 2.2 Hz, H-4’). The methoxyl signal, identical at *δ_H_* 3.57 (6H, s), was attributed to 3’, 5’-OCH_3_. A –CH_2_–CH–CH– structural unit could be revealed by the peak splitting and coupling constants of the proton signals at *δ_H_* [2.66 (1H, dd, *J* = 17.4, 8.8 Hz) and 2.52 (1H, dd, *J* = 17.4, 2.8 Hz), H-3], 4.07 (1H, ddd, *J* = 8.8, 3.3, 2.8 Hz, H-4), and 5.96 (1H, d, *J* = 3.3 Hz, H-5). The fragments were confirmed by the ^1^H–^1^H COSY correlations of **4**. The ^13^C NMR of **4** showed an unconjuated ketone carbonyl at *δ_C_* 207.8. The correlation between *δ_H_* 4.85 (1H, s, H-1) and *δ_C_* 79.7 (C-1), found in the HSQC spectrum, revealed an oxygen-linked methylidyne. The HMBC correlations between *δ_H_* 4.85 (H-1) and *δ_C_* 141.7 (C-1’), 207.8 (C-2), and between *δ_H_* 2.66 and 2.52 (H-3) and *δ_C_* 207.8 (C-2), allowed the elucidation of the structure of **4** (Figure 2).

We isolated 2,3,4-trihydroxybutyl cinnamate (**5**) as a white powder. The molecular formula of C_13_H_16_O_5_ was revealed by the HR-ESI-MS cationized ion peaks at *m*/*z* 275.0882 [M + Na]^+^ (calcd. for 275.0890 C_13_H_16_O_5_Na) in positive mode, and *m*/*z* 251.0922 [M − H]^−^ (calcd. for 251.0925, C_13_H_14_O_5_) in negative mode. A conjugated ester carbonyl IR signal was present at 1700 cm^−1^. A *mono*-substituted benzene ring was deduced by the ^1^H NMR signal at *δ_H_* 7.72 (2H, dd, *J* = 6.3, 3.0 Hz, H-2, 6) and *δ_H_* 7.74 (3H, overlapped, H-3,5 and H-4). A pair of *trans*-alkene proton signals were exhibited at *δ_H_* 7.69 (1H, d, *J* = 16.0 Hz, H-7) and *δ_H_* 6.64 (1H, d, *J* = 16.0 Hz, H-8). With the help of ^1^H–^1^H COSY spectrum, two ABX spin systems at *δ_H_* [4.33 (1H, dd, *J* = 11.3, 2.7 Hz) and 4.11 (1H, dd, *J* = 11.3, 7.1 Hz), H-1′], 3.66 (1H, m, H-2′), and at *δ_H_* [3.58 (1H, br. d, *J* = 9.2 Hz) and 3.42 (1H, br. d, *J* = 9.2 Hz), H-4′], 3.44 (1H, m, H-3′), were connected to a structure fragment as O–CH_2_–CH(O)–CH(O)–CH_2_–O (Figure 2). In the HMBC spectrum, correlations from H-1’ (*δ_H_* 4.33 & 4.11), H-8 (*δ_H_* 6.64) to C-9 (*δ_C_* 166.9), and from H-3, 5 (*δ_H_* 7.44), H-8 (*δ_H_* 6.64) to C-1 (*δ_C_* 134.5), allowed the elucidation of 2,3,4-trihydroxybutyl cinnamate. The chlorogenic acid contained in the potato medium may be the original precursor of this compound.

The structures of compounds **6**–**24** were elucidated by the comparison of their MS and NMR data with those reported in the literature, and they were identified as: 4-methyl-5,6-dihydropyren-2-one (**6**) [15], citreo-g-pyrone (**7**) [16], pyrenocine B (**8**) [17], pyrenocine D (**9**) [18], pyrenocine A (**10**) [19], pyrenocine I (**11**) [20], pyrenocine E (**12**) [18], pyrenocine J (**13**) [21], citreovirenone (**14**) [12], citroethiolactone (**15**) [16], citreoviridin A (1**6**) [22], isocitreoviridin A (**17**) [22], aurovertin U (**18**) [14], cyclo (*phe*-*trp*) (**19**) [14], *N*-(*N*-acetyl-valyl)-phenylalanine (**20**) [23], 2′,3′-dihydrosorbicillin (**22**) [24], citreoviranol (**23**) [25], and haenamindole (**24**) [26]. Most of the compounds have been isolated from the genus of *Penicillum.* Compound **21**, a known compound without reported NMR data, was identified by MS and 1D, 2D NMR spectra data as 3,5-dihydroxy-2,4-dimethyl-6-(3-oxobutan-2-yl)benzaldehyde.

### 2.2. Cytotoxicity Evaluation

All of the isolated compounds were evaluated for their cytotoxic effects on four types of tumor cells; Bel7402, HT1080, CNE2 and A549. Compounds **2** and **4** showed potent cytotoxicity to the cell lines Bel7402 and HT1080, while they showed no obvious effects on Cne2 and A549 (Table 2). All other compounds exhibited much lower cytotoxicity to the four tumor cell lines, with IC_50_ values larger than 100 μM. 

## 3. Discussion

In this study, five new compounds, including two new heteroatom-containing compounds were isolated from the ethyl acetate extract of a deep-sea fungus *P. citreonigrum* XT20-134 (MCCC 3A00956). Chemically, the relative configuration of these compounds was confirmed by their NOESY spectra; the absolute configuration of compound **1** was revealed by comparison of its CD spectra with the calculated ECD. All of the compounds were evaluated for cytotoxic activity. The new heteroatom-containing compounds **2** and **4** showed potent cytotoxicity to the tumor cell lines Bel7402 and HT1080. 

Marine microorganisms can utilize chloride ions or sulfate ions, the two most abundant anions in seawater, and produce heteroatom-containing compounds, with diverse chemical structures and various bioactivities [27]. The bioactive halogenated, mainly referring to chlorinated, natural products from microorganisms mainly manifest with cytotoxic and antibacterial activity, indicating that halogenated compounds produced from microorganisms, due to adaptability or defense from the extreme environment, have cytotoxic and antibacterial activity [28]. In this study, compound **4** that contained two choline atoms was also found to have potent cytotoxicity. The most abundant source of sulfur-containing natural products is also marine organisms. Sulfur can appear in a multitude of combinations and oxidation states: thiol, sulfide, disulfide, sulfoxide, sulfonate, etc. The diversity of sulfur-containing chemical structures leads to their various bioactivities [29]. Among them, psammaplin A, has been found to have a broad bioactive spectrum, especially in terms of antimicrobial and antiproliferative activities [30]. With a sulfur atom, compound **2** exhibited potent cytotoxic activity. Similar compounds without sulfur (**1**, **8**, and **10**) did not shown cytotoxic activity. However, compound **16** show no activity, indicating that the type of sulfur bond is important to the activity. 

## 4. Materials and Methods 

### 4.1. General Experimental Procedures

An electrospray ionization source (ESI)-equipped Q-Exactive mass spectrometer (Thermo Fisher Scientific Corporation, Waltham, MA, USA) was used to analyze the HR-ESI-MS data. A Shimadzu UV-260 spectrometer (Shimadzu Corporation, Tokyo, Japan) and a Perkin-Elmer 683 infrared spectrometer (PerkinElmer, Inc., Waltham, MA, USA) were used to obtain the UV and IR spectra, respectively. A JASCO P-200 polarimeter (JASCO Corporation, Tokyo, Japan) with a 5 cm cell was applied to measure the optical rotation value. The NMR spectra with TMS as the internal standard were taken on a Brucker Avance III 600 FT NMR spectrometer (Bruker Corporation, Billerica, MA, USA). 

### 4.2. Eletronic Circular Dichroism (ECD) Calculations

The theoretical electronic circular dichroism (ECD) spectra of the isolated compounds were calculated on the basis of the relative configurations determined by their NOESY spectra and *J* values in ^1^H NMR. Conformational analyses and density functional theory (DFT) calculations were used to generate and optimize the conformers with energy. The ECD calculations were performed as previously described [31,32].

### 4.3. Fungal Strain and Fermentation

The strain *Penicillium* sp. XT20-134 was isolated from southeast Indian Ocean sediments at 2910 m by the tablet pour method. The internal transcribed spaces (ITS) region was amplified and sequenced using the general primers ITS1 and ITS4. The ITS region of the fungi is a 573 bp DNA sequence (GenBank Accession Number: KY 978587) that showed 99% identity to *P. citreonigrum*. The strain was deposited at the China Center for Type Culture Collection (CCTCC) as accession number M2017125 and Marine Culture Collection of China (MCCC) as accession number MCCC 3A00956. The fungus grew well on the rice medium in artificial seawater. Carbohydrate fermentation was conducted by subculturing the fungus in rice medium in artificial seawater and incubating at 28 °C for 30 days in a standing position.

### 4.4. Extraction and Isolation

The rice medium (10 kg) of *P. citreonigrum* XT20-134 was extracted with ethyl acetate (20 L) trice and concentrated under reduced pressure at 40 °C to yield 19.2 g of the residue.

The EtOAc extract (18 g) was fractionated over a column packed with silica gel (360 g, Yantai Chemical Industry Research Institute, Yantai, China), eluted with petroleum ether-ethyl acetate (*v*/*v*) (5:1, 1.0 L) and CHCl_3_–CH_3_OH (*v*/*v*) (5;1, 1.0 L) and CH_3_OH (1.0 L), to afford **PE** eluent (0.5 g), **CM** eluent (12.3 g), and methanol eluent (3.4 g). The **PE** eluent was purified by a silica gel (30 g) column and eluted with petroleum ether-ethyl acetate (*v*/*v*) (10:1, 5:1, and 3:1, each 200 L) to give compound **22** (13 mg). The **CM** eluent was separated over a Cosmosil reversed-phase C18 (300 g, 75 μm, Nakalai Tesque Co. Ltd., Kyoto, Japan) column and eluted with CH_3_OH/H_2_O (10%–100%, each 1.5 L) to provide 12 fractions (Fr. 1–Fr. 12). Compound **24** (83 mg) was obtained from Fr. 11 after recrystallization. Other fractions were purified over a preparative Cosmosil ODS column (250 mm × 20.0 mm i.d., 5 μm, Cosmosil, Nakalai Tesque Co. Ltd., Kyoto, Japan), and isocratically eluted with a mobile phase system of acetonitrile–H_2_O in different ratios. Eluting with 15% acetonitrile, preparative HPLC separation on Fr. 1, Fr. 2, and Fr. 3 resulted in the isolation of: compound **6** (6 mg) from Fr. 1, compounds **7** (8 mg), **8** (57 mg), **9** (5 mg) from Fr. 2, and compounds **1** (50 mg), **10** (90 mg), **11** (15 mg), **12** (8 mg) from Fr. 3, respectively. Preparative HPLC purification of Fr. 5, eluted with acetonitrile–H_2_O (30:70, *v*/*v*), led to the isolation of compound **2** (15 mg) and compound **15** (150 mg). Fr. 4, Fr. 6, and Fr. 7 were separated with 30% acetonitrile. As a result, compounds **5** (5 mg), **13** (4 mg), and **14** (5 mg) were isolated from Fr. 4; compound **19** (6 mg) from Fr. 6, and compounds **20** (13 mg) and **21** (20 mg) from Fr. 7, respectively. Fr. 8 was isolated with 35% acetonitrile to obtain compound **4** (4 mg). Separation of Fr. 9 and Fr. 10, eluted with 40% acetonitrile, obtained compound **3** (8 mg) and compound **23** (13 mg), respectively. The other three compounds, **16** (15 mg), **17** (18 mg) and **18** (13 mg), were obtained from Fr. 12, by eluting with 45% acetonitrile.

### 4.5. Structrural Elucidation of the New Compounds **1**–**5**

Adeninylpyrenocine (**1**): white amorphous powder; [α${]}_{D}^{25}$ − 14° (c = 0.1, CH_3_OH); IR (KBr) (ν_max_): 3441, 1695, 1642, 1402 and 1256 cm^−1^; UV (MeOH) λ_max_ (log *ε*): 204 (2.49) and 261 (2.22) nm. HR-ESI-MS: *m*/*z* 344.1357 [M + H]^+^ (calcd. for 344.1353 C_16_H_18_N_5_O_4_) and 366.1172 [M + Na]^+^ (calcd. for 366.1173 C_16_H_17_N_5_O_4_Na) in positive mode, and *m*/*z* 342.1220 [M − H]^−^ (calcd. for 342.1208, C_16_H_16_N_5_O_4_) in negative mode. ^1^H NMR (600 MHz, DMSO-*d_6_*) and ^13^C NMR (150 MHz, DMSO-*d_6_*) spectra data are listed in Table 1, and Figures S1–S9 in supporting materials.

2-Hydroxyl-3-pyrenocine-thio propanoic acid (**2**): light yellow powder; [α${]}_{D}^{25}$ + 26° (c = 0.1, CH_3_OH); IR (KBr) (ν_max_): 3435, 2927, 1714, 1626, 1453, 1400, 1260, and 1096 cm^−1^; UV (MeOH) λ_max_ (log *ε*): 203 (2.10) nm and 260 (2.52) nm. HR-ESI-MS: *m*/*z* 331.0845 [M + H]^+^ (calcd. for 331.0859 C_14_H_19_O_7_S) and 353.0670 [M + Na]^+^ (calcd. for 353.0665 C_14_H_18_O_7_SNa) in positive mode, and *m*/*z* 329.0706 [M − H]^−^ (calcd. for 329.0700, C_14_H_17_O_7_S) in negative mode. ^1^H NMR (600 MHz, DMSO-*d_6_*) and ^13^C NMR (150 MHz, DMSO-*d_6_*) spectra data are listed in Table 1, and Figures S10–S17 in supporting materials.

Ozazino-*cyclo*-(2,3-dihydroxyl-trp-tyr) (**3**): white powder; [α${]}_{D}^{25}$ + 43° (c = 0.1, CH_3_OH); IR (KBr) (ν_max_): 3437, 1669, 1623, 1460, 1396, and 1081 cm^−1^; UV (MeOH) λ_max_ (log *ε*): 204 (2.55) nm, 240 (1.92) nm, and 299 (1.55) nm. HR-ESI-MS: *m*/*z* 366.1449 [M + H]^+^ (calcd. for 366.1448 C_20_H_20_N_3_O_4_) and 388.1265 [M + Na]^+^ (calcd. for 388.1268 C_20_H_19_N_3_ONa) in positive mode, and *m*/*z* 364.1303 [M − H]^−^ (calcd. for 364.1303, C_20_H_18_N_3_O) in negative mode. ^1^H NMR (600 MHz, DMSO-*d_6_*) and ^13^C NMR (150 MHz, DMSO-*d_6_*) spectra data are listed in Table 3, and Figures S18–S25 in supporting materials.

5,5-Dichloro-1-(3,5-dimethoxyphenyl)-1,4-dihydroxypentan-2-one (**4**): white powder; [α${]}_{D}^{25}$ − 23° (c = 0.1, CH_3_OH); IR (KBr) (ν_max_): 3447, 1608, 1396, and 1159 cm^−1^; UV (MeOH) λ_max_ (log *ε*): 204 (2.36) nm and 275 (1.23) nm. HR-ESI-MS: *m*/*z* 323.0449 [M + H]^+^ (calcd. for 323.0448 C_13_H_17_O_5_Cl_2_) and 345.0261 [M + Na]^+^ (calcd. for 345.0267 C_13_H_16_O_5_Cl_2_Na) in positive mode, and *m*/*z* 321.0304 [M − H]^−^ (calcd. for 321.0302, C_13_H_15_O_5_Cl_2_) in negative mode. ^1^H NMR (600 MHz, DMSO-*d_6_*) *δ_H_*: 6.37 (2H, d, *J* = 2.2 Hz, H-2′, 6′), 6.27 (1H, t, *J* = 2.2 Hz, H-4′), 5.96 (1H, d, *J* = 3.3 Hz, H-5), 4.85 (1H, s, H-1), 4.07 (1H, ddd, *J* = 8.8, 3.3, 2.8 Hz, H-4), 3.57 (6H, s, H-3′, 5′-OCH_3_), [2.66 (1H, dd, *J* = 17.4, 8.8 Hz) & 2.52 (1H, dd, *J* = 17.4, 2.8 Hz), H-3]; ^13^C NMR (150 MHz, DMSO-*d_6_*) *δ_C_*: 207.8 (C-2), 160.9 (C-3′, 5′), 141.7 (C-1′), 105.3 (C-2′, 6′), 100.0 (C-4′), 79.7 (C-1), 77.7 (C-5), 71.1 (C-4), 55.6 (C-3′, 5′-OCH_3_), 40.9 (C-3). The spectra are provided in Figures S26–S33 in supporting materials.

2,3,4-Trihydroxybutyl cinnamate (**5**): white powder; [α${]}_{D}^{25}$ − 23° (c = 0.1, CH_3_OH); IR (KBr) (ν_max_): 3421, 1700, 1634, 1396, 1186 and 1085 cm^−1^; UV (MeOH) λ_max_ (log *ε*): 216 (1.93) nm and 276 (1.99) nm. HR-ESI-MS: *m*/*z* 275.0882 [M + Na]^+^ (calcd. for 275.0890 C_13_H_16_O_5_Na) in positive mode, and *m*/*z* 251.0922 [M − H]^−^ (calcd. for 251.0925, C_13_H_14_O_5_) in negative mode. ^1^H NMR (600 MHz, DMSO-*d_6_*) *δ_H_*: 7.69 (1H, d, *J* = 16.0 Hz, H-7), 7.44 (1H, overlapped, H-4), 7.44 (2H, overlapped, H-3, 5), 7.72 (2H, dd, *J* = 6.3, 3.0 Hz, H-2, 6), 6.64 (1H, d, *J* = 16.0 Hz, H-8), 3.44 (1H, m, H-3′), 3.66 (1H, m, H-2′), [4.33 (1H, dd, *J* = 11.3, 2.7 Hz) and 4.11 (1H, dd, *J* = 11.3, 7.1 Hz), H-1′], [3.58 (1H, br. d, *J* = 9.2 Hz) and 3.42 (1H, br. d, *J* = 9.2 Hz), H-4′]; ^13^C NMR (150 MHz, DMSO-*d_6_*) *δ_C_*: 166.9 (C-9), 144.8 (C-7), 134.5 (C-1), 130.9 (C-4), 129.4 (C-3, 5), 128.8 (C-2, 6), 118.8 (C-8), 72.8 (C-3′), 69.9 (C-2′), 67.0 (C-1′), 63.5 (C-4′). The spectra are provided in Figures S34–S42 in supporting materials.

### 4.6. Cytotoxicity Assay

Cytotoxicity assay was carried out according to the instructions of CCK-8 kit [7]. Briefly, compounds at different concentrations were added into the culture medium containing 10^5^ /mL HT1080, Cne2 and Bel7402 cells and incubated for 24, 48, 72 h. Then, 10 μL of CCK-8 solution was added into each well of the 96-well plate, incubated for 2 h and then the absorbance was measured at 450 nm using a microplate reader (BIO-RAD, Hercules, California, USA). The inhibition rate = (A _control_ − A _treated_)/A _control_ × 100. The IC_50_ was the concentration at which it caused 50% inhibition of cell proliferation (50% reduction in the absorbance value in the treated cells, in respect to the control).

## Figures and Tables

**Table 1:** ^1^H NMR and ^13^C NMR (DMSO-*d*_6_) data of compounds **1** and **2**.

Positions	1		2		2’	
	*δ_C_*	*δ_H_*	*δ_C_*	*δ_H_*	*δ_C_*	*δ_H_*
2	162.0		162.2		162.2	
3	87.8	5.62, s	87.9	5.69, s	87.9	5.69, s
4	168.3		168.5		168.5	
5	115.1		115.4		115.4	
6	162.8		163.2		163.2	
7	198.8		199.5		199.5	
8	49.3	3.78, dd (17.6, 7.9)	51.7	2.99, dd (17.1, 6.6)	51.6	2.99, dd (17.1, 6.6)
		3.35, dd (17.6, 6.0)		2.90, dd (17.1, 3.7)		2.89, dd (17.1, 3.7)
9	47.7	5.06, m	36.3	3.30, m	36.2	3.30, m
10	21.1	1.54, br. d (6.6)	21.8	1.23, d (6.8)	21.8	1.23, d (6.8)
6-CH_3_	18.0	1.85, s	18.5	2.18, d (2.6)	18.5	2.18, d (2.6)
4-OCH_3_	57.4	3.74, s	57.5	3.87, s	57.5	3.87, s
2′	152.6	8.11, br. s				
4′	149.5					
5′	119.5					
6′	156.4					
8′	140.3	8.21, br. s				
6′-NH_2_		7.16, br. s				
1′			174.5		174.5	
2′			71.1	4.09, m	71.0	4.09, m
3′			34.7	2.83, dd (13.5, 5.1)	34.6	2.82, dd (13.5, 5.1)
				2.72, dd (13.6, 9.5)		2.71, dd (13.6, 9.5)

**Table 2:** Cytotoxic activities of compounds **2** and **4** (IC_50_, μM).

Compd.	Bel7402	HT1080	Cne2	A549
**2**	7.63 ± 1.46	10.22 ± 1.32	73.14 ± 5.32	87.08 ± 7.32
**4**	13.14 ± 1.41	16.53 ± 1.67	83.56 ± 6.49	92.47 ± 6/33
paclitaxel	<1	<1	<1	<1
DMSO	None	None	None	None

**Table 3:** ^1^H NMR and ^13^C NMR (DMSO-*d*_6_) data of compounds **3** and **19**.

Positions	3		19	
	*δ_C_*	*δ_H_*	*δ_C_*	*δ_H_*
2	99.3	5.10, d (2.8)	121.4	6.88, br. s
3	74.6		109.2	
4	123.1	6.90, br. d (7.2)	118.9	7.43, br. d (7.2)
5	118.3	6.56, br. t (7.0)	119.2	6.93, br. t (7.0)
6	129.1	7.00, br. t (7.5)	124.9	7.01, br. t (7.4)
7	109.6	6.56, br. d (7.7)	111.8	7.26, br. d (7.7)
3a	131.9		128.0	
7a	148.1		136.5	
1′	136.0		137.0	
2′, 6′	130.4	6.98, br. d (7.2)	130.2	7.10, overlapped
3′, 5′	128.2	6.86, br. t (7.4)	128.5	6.64, br. t (7.4)
4′	126.5	6.75, m	126.9	7.12, overlapped
C=O	166.1		166.7	
C=O′	161.5		167.3	
α	54.6	4.17, dd (9.1, 4.8)	55.8	3.92, m
α′	54.8	4.19, dd (5.0, 2.8)	56.1	3.79, m
β′	37.7	2.87, dd (13.9, 2.8)	39.5	2.40, overlapped
		2.74, dd (13.8, 5.0)		1.78, dd (13.5, 7.0)
β	36.0	1.91, dd (13.6, 4.8)	30.2	2.74, dd (14.0, 4.4)
		1.03, dd (13.5, 9.1)		2.44, overlapped
1-NH		6.57, br. s		10.83, br. s
α-NH				7.85, br. s
α′-NH		7.96, br. s		7.64, br. s

## Supplementary Materials

The following are available online at https://www.mdpi.com/1660-3397/17/9/509/s1, Figures S1–S42: Supporting NMR, IR, and HR-ESI-MS spectra.
