# Supplementary material for: Two New Cytotoxic Compounds from a Deep-Sea Penicillum citreonigrum XT20-134
Source: Mar Drugs. 2019 Aug 29;17(9):509. doi: 10.3390/md17090509 (PMC6780507; doi:10.3390/md17090509)
Supplement: Supplementary file 1 [file marinedrugs-17-00509-s001.pdf]

Two New Cytotoxic Compounds from a Deep-sea *Penicillum citreonigrum* XT20-134

**Content**	Page
Figure S 1. IR spectrum of compound **1**	3
Figure S 2. HR-ESI-MS spectra of compound **1**	4
Figure S 3. ^{1}H-NMR (600 MHz, DMSO-d6) of compound **1**	5
Figure S 4. ^{13}C-NMR (150 MHz, DMSO-d6) of compound **1**	6
Figure S 5. DEPT of compound **1**	7
Figure S 6. HSQC of compound **1**	8
Figure S 7. ^{1}H-^{1}H COSY of compound **1**	9
Figure S 8. HMBC of compound **1**	10
Figure S 9. NOESY of compound **1**	11
Figure S 10. IR spectrum of compound **2**	12
Figure S 11. HR-ESI-MS spectra of compound **2**	13
Figure S 12. ^{1}H-NMR (600 MHz, DMSO-d6) of compound **2**	14
Figure S 13. ^{13}C-NMR (150 MHz, DMSO-d6) of compound **2**	15
Figure S 14. DEPT of compound **2**	16
Figure S 15. HSQC of compound **2**	17
Figure S 16. ^{1}H-^{1}H COSY of compound **2**	18
Figure S 17. HMBC of compound **2**	19
Figure S 18. IR spectrum of compound **3**	20
Figure S 19. HR-ESI-MS spectra of compound **3**	21
Figure S 20. ^{1}H-NMR (600 MHz, DMSO-d6) of compound **3**	22
Figure S 21. ^{13}C-NMR (150 MHz, DMSO-d6) of compound **3**	23
Figure S 22. DEPT of compound **3**	24
Figure S 23. HSQC of compound **3**	25
Figure S 24. ^{1}H-^{1}H COSY of compound **3**	26
Figure S 25. HMBC of compound **3**	27
Figure S 26. IR spectrum of compound **4**	28
Figure S 27. HR-ESI-MS spectra of compound **4**	29
Figure S 28. ^{1}H-NMR (600 MHz, DMSO-d6) of compound **4**	30
Figure S 29. ^{13}C-NMR (150 MHz, DMSO-d6) of compound **4**	31
Figure S 30. DEPT of compound **4**	32
Figure S 31. HSQC of compound **4**	33
Figure S 32. ^{1}H-^{1}H COSY of compound **4**	34
Figure S 33. HMBC of compound **4**	35
Figure S 34. IR spectrum of compound **5**	36
Figure S 35. HR-ESI-MS spectra of compound **5**	37
Figure S 36. ^{1}H-NMR (600 MHz, DMSO-d_6) of compound **5**	38
Figure S 37. ^{13}C-NMR (150 MHz, DMSO-d_6) of compound **5**	39
Figure S 38. DEPT of compound **5**	40
Figure S 39. HSQC of compound **5**	41
Figure S 40. ^{1}H-^{1}H COSY of compound **5**	42

Figure S 41. HMBC of compound **5** 43
Figure S 42. Colony morphology photograph of *Penicillum citreonigrum* XT20-134. 44

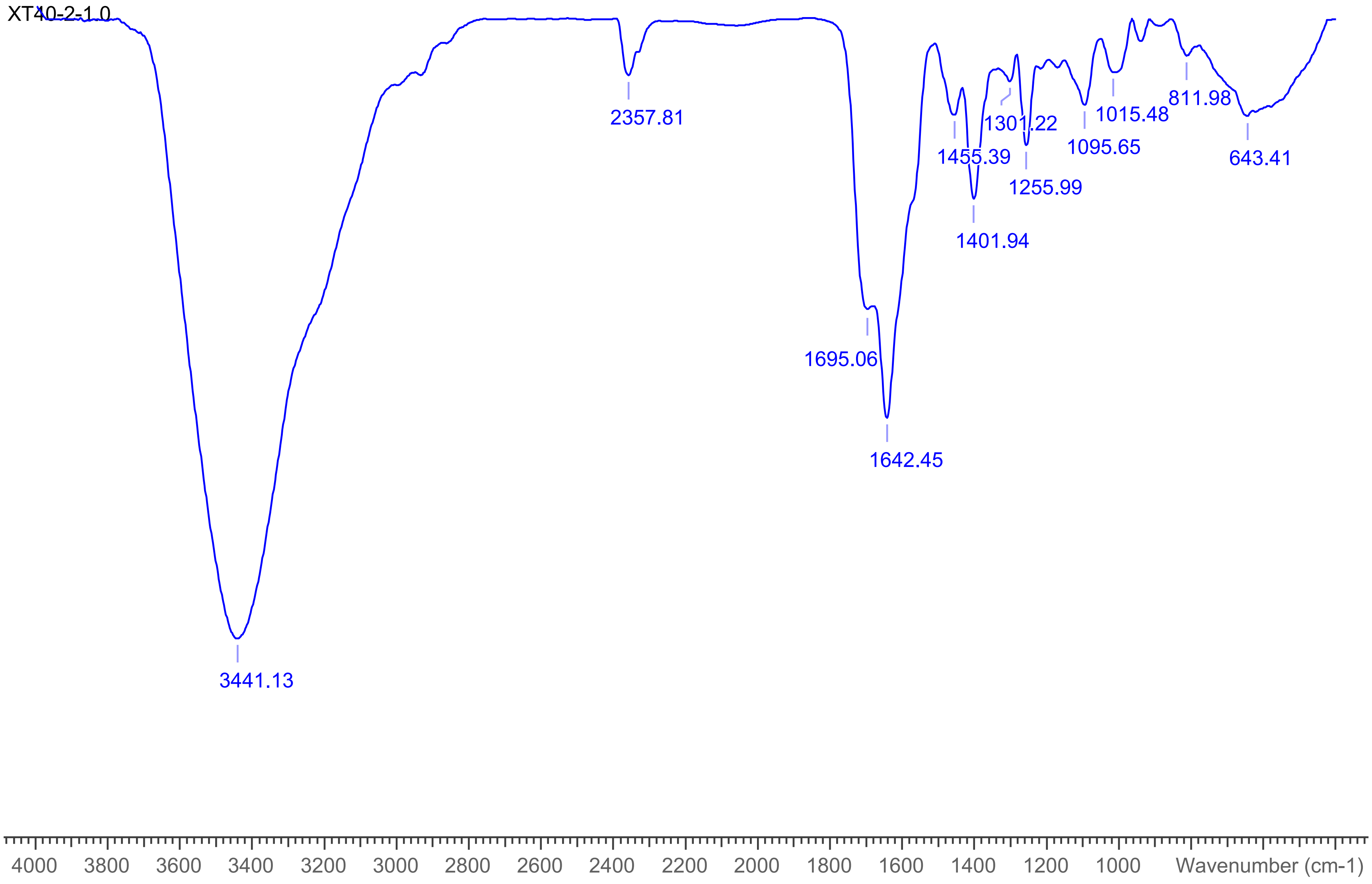

Figure S 1. IR spectrum of compound **1**

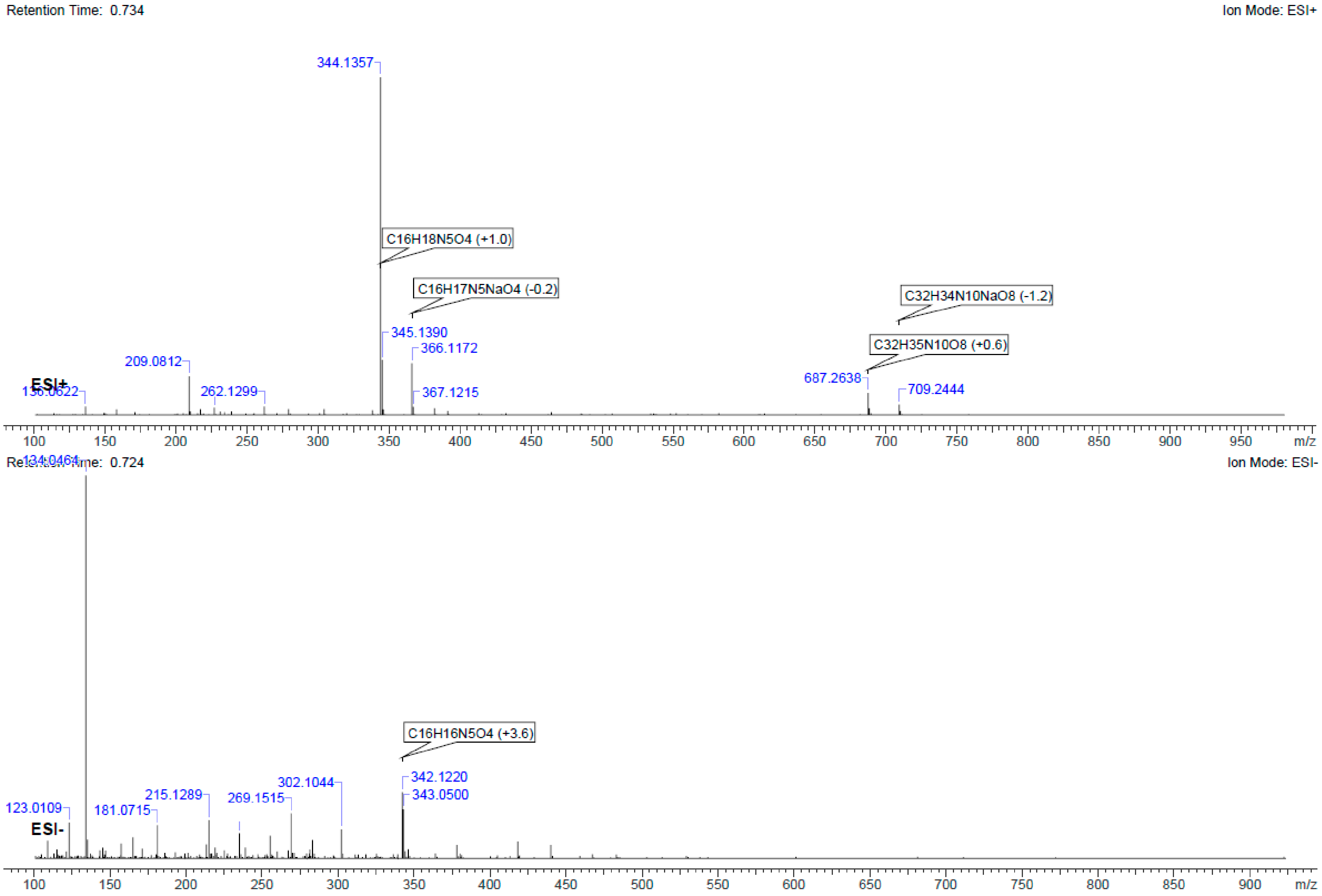

Figure S 2. HR-ESI-MS spectra of compound **1**

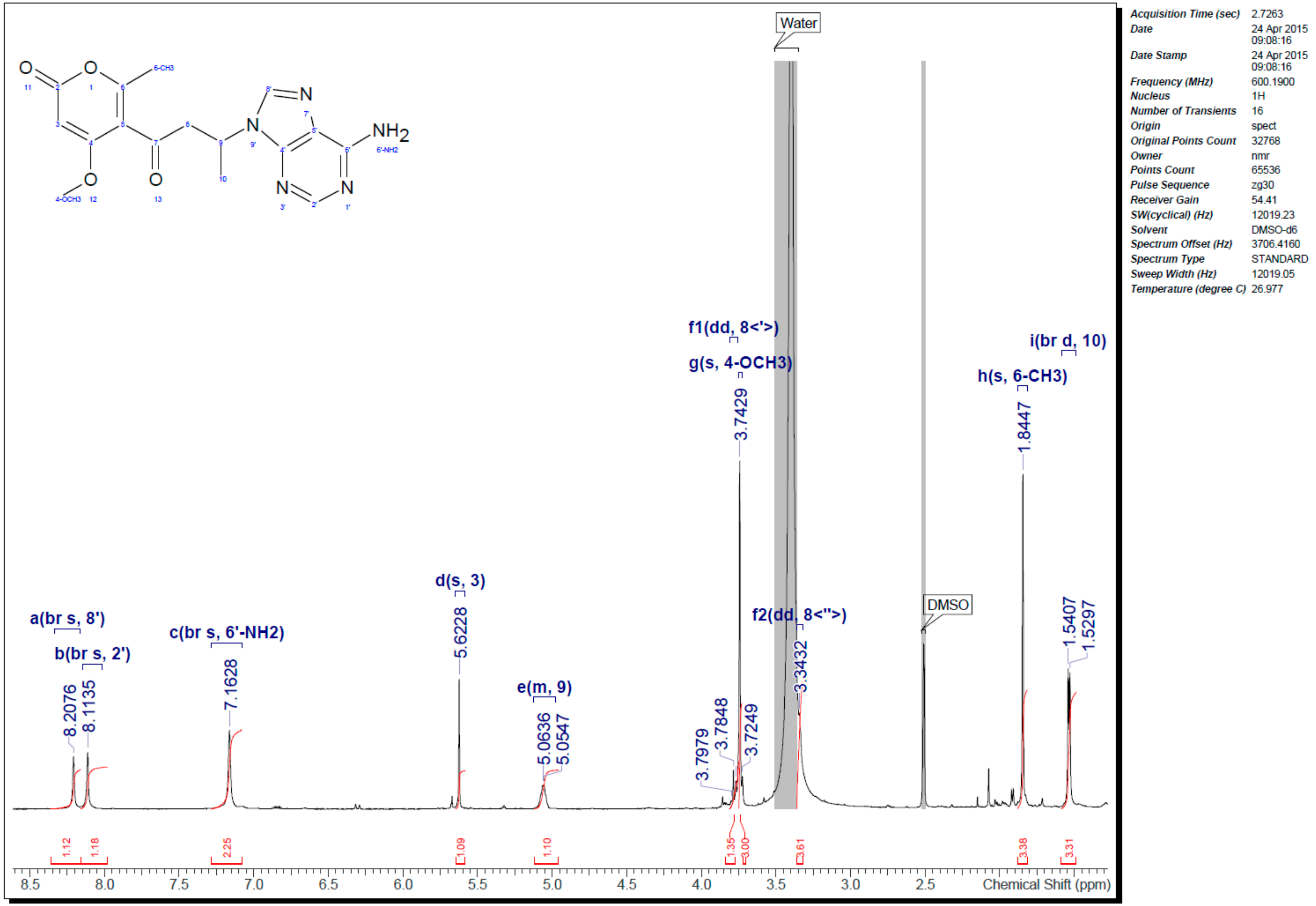

Figure S 3. ^{1}H-NMR (600 MHz, DMSO-d_6) of compound **1**

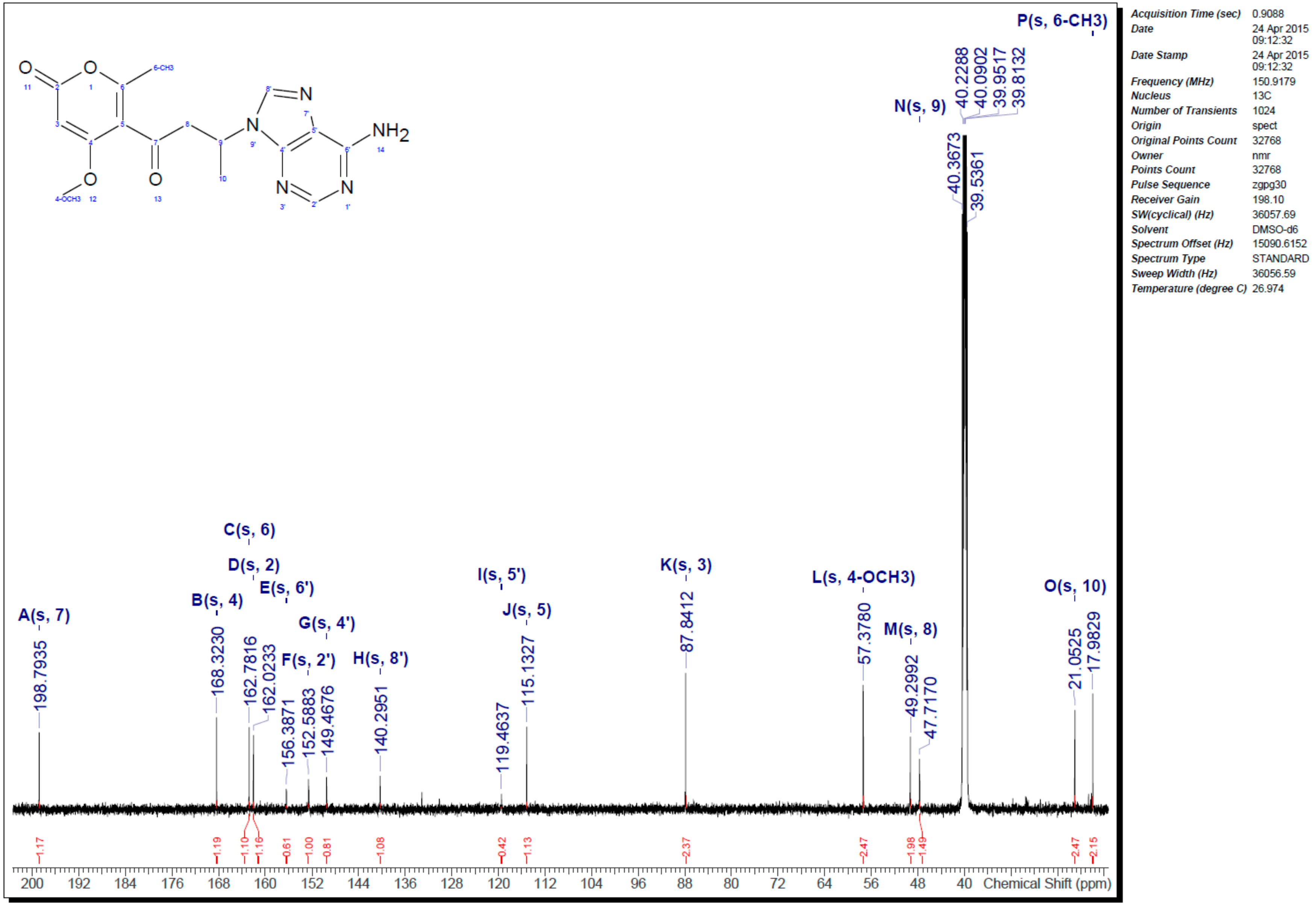

Figure S 4. ^{13}C-NMR (150 MHz, DMSO-d_6) of compound **1**

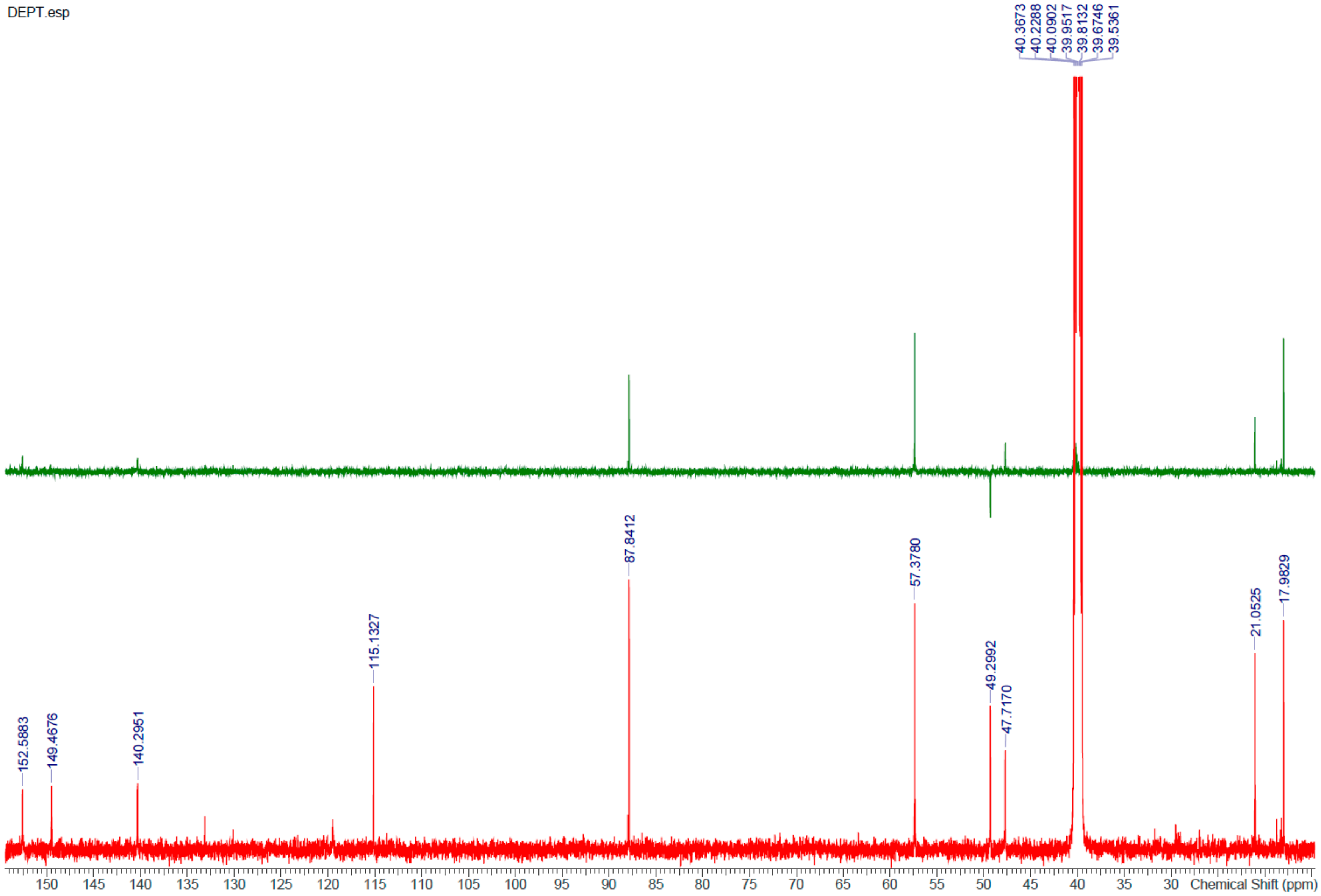

Figure S 5. DEPT of compound **1**

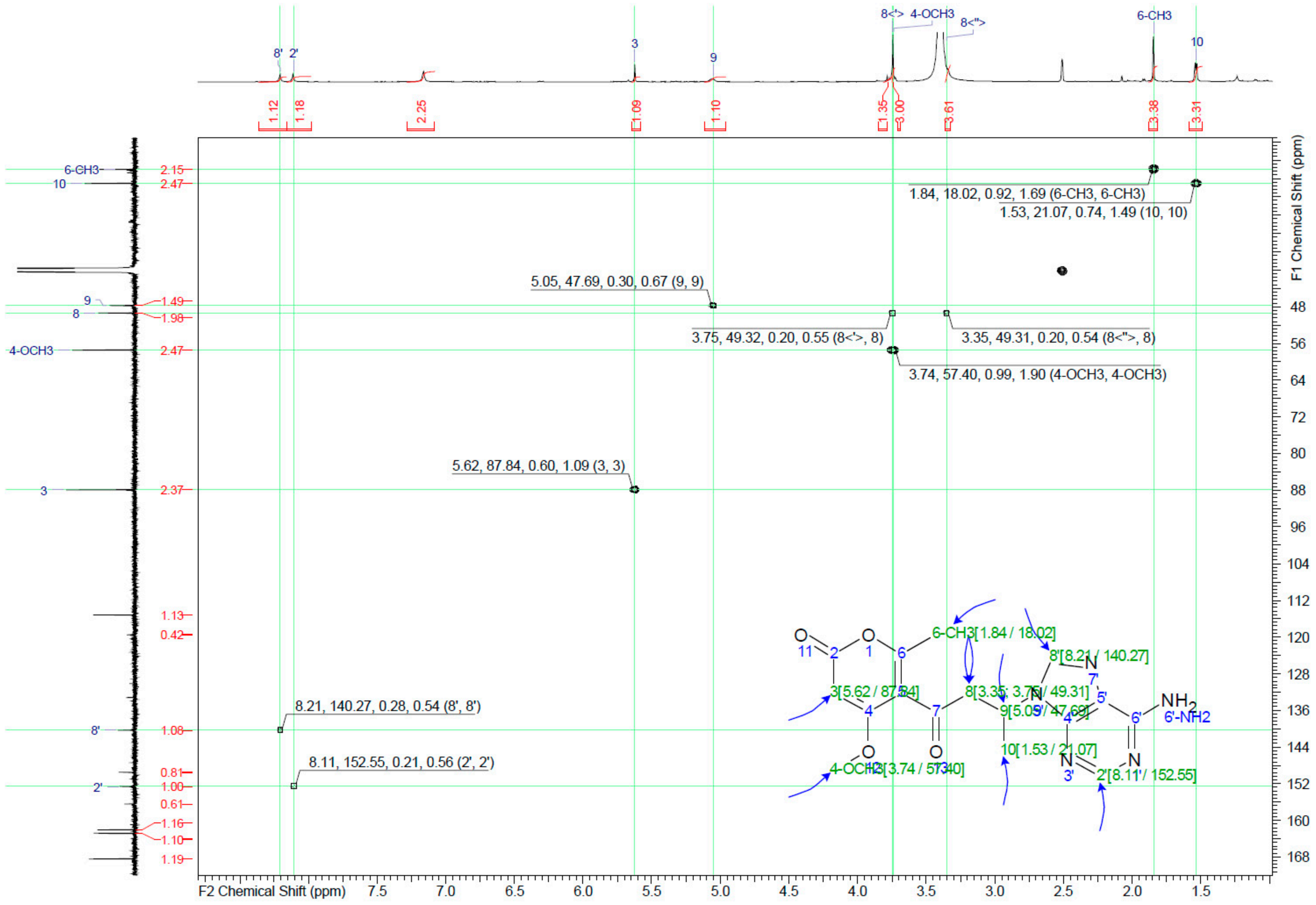

Figure S 6. HSQC of compound **1**

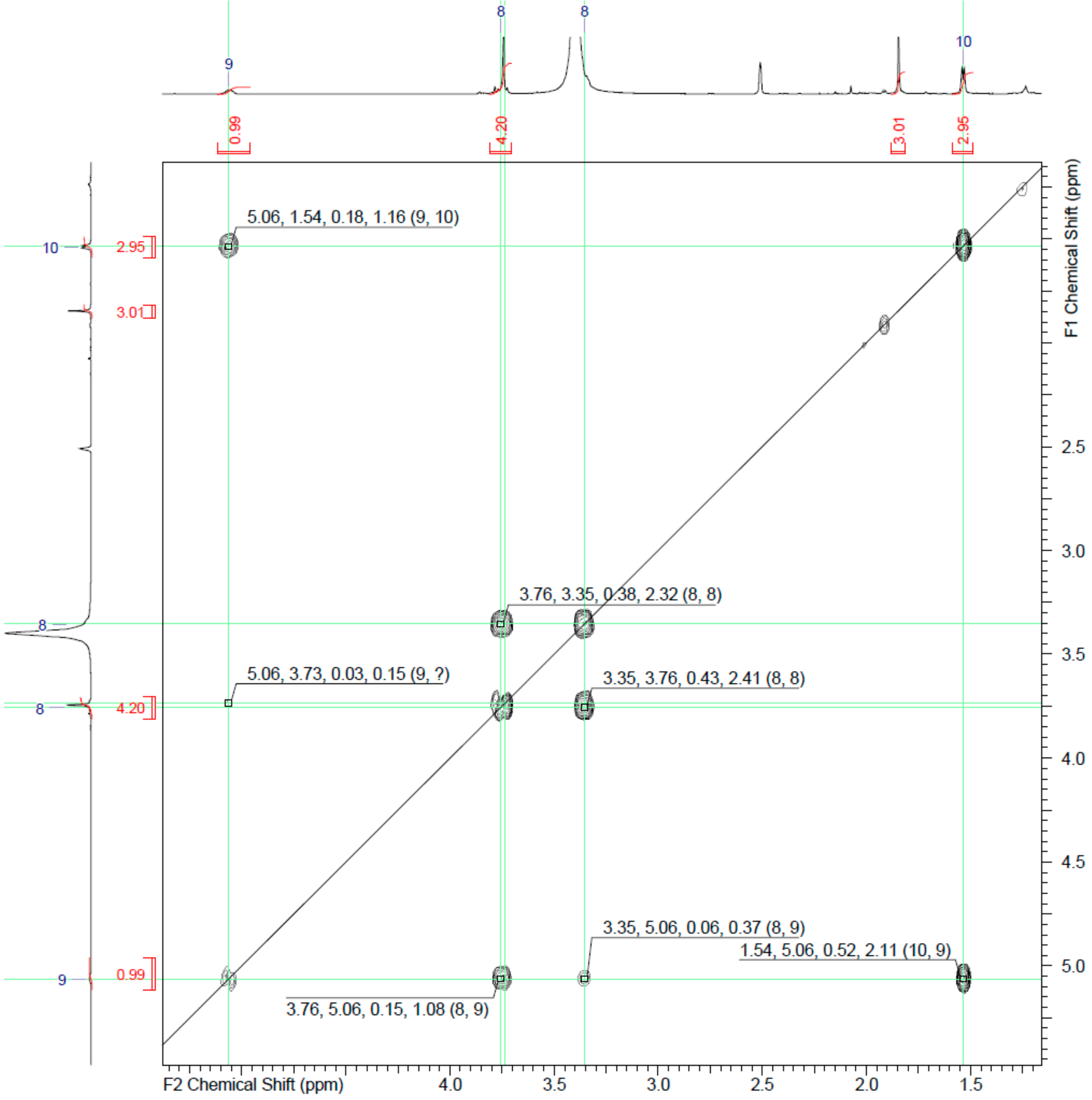

Acquisition Time (sec)	(0.0852, 0.0214)
Comment	5 mm PABBO BB/19F-1H/D Z-GRD Z114607/0133
Date	22 Dec 2016 03:20:48
File Name	C:\Users\Administrator\Desktop\yanxia-20161222\XT40%-3-1\4\pdata\1\2rr
Frequency (MHz)	(600.1936, 600.1936)
Nucleus	(1H, 1H)
Number of Transients	4
Origin	spect
Original Points Count	(1024, 256)
Owner	nmr
Points Count	(2048, 1024)
Pulse Sequence	cosygpmfqf
Solvent	DMSO-d6
Spectrum Type	COSY
Sweep Width (Hz)	(12013.36, 11978.70)
Temperature (degree C)	23.964
Title	

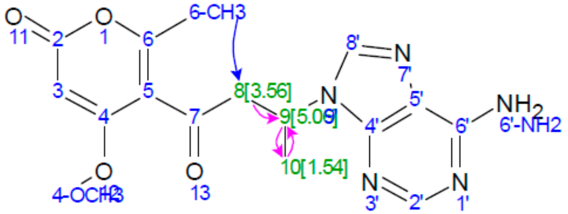

Figure S 7. ^{1}H-^{1}H COSY of compound **1**

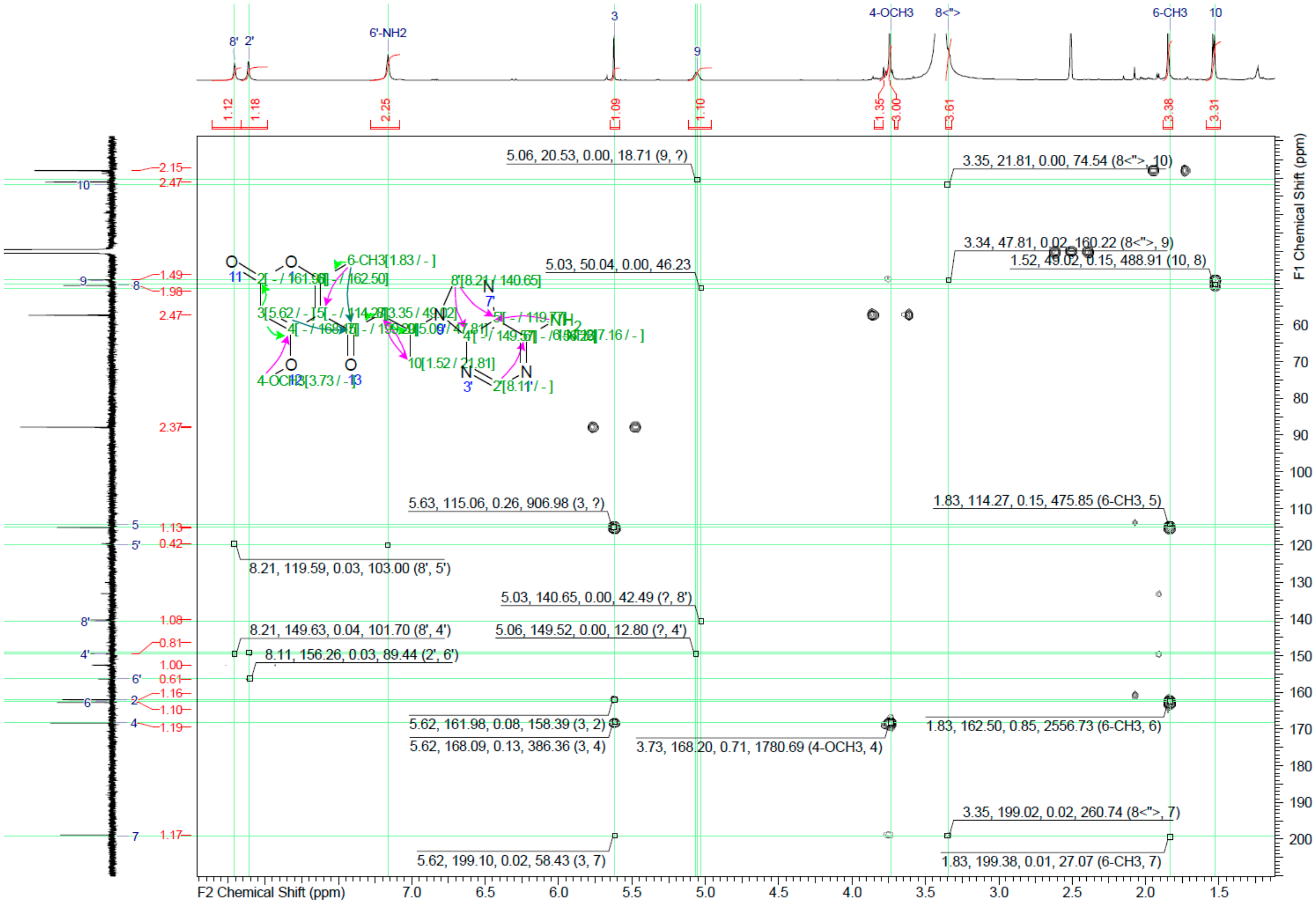

Figure S 8. HMBC of compound **1**

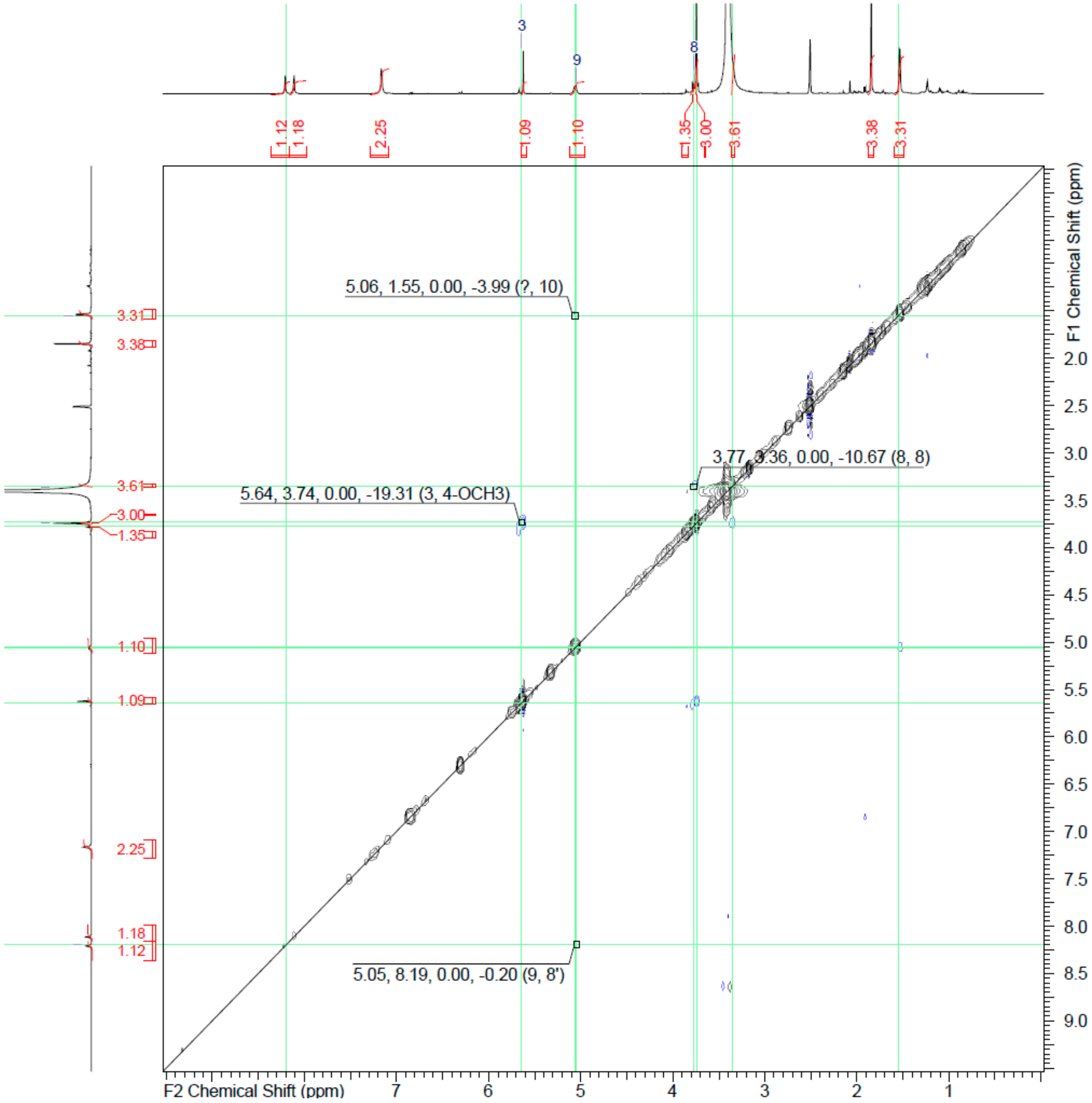

Acquisition Time (sec)	(0.1782, 0.0445)
Comment	5 mm PABBO BB/19F-1H/D Z-GRD Z114607/0133
Date	22 Dec 2016 09:37:14
File Name	C:\Users\Administrator\Desktop\yanxia-20 161222\XT40%-3-1\7\pdata\1\2rr
Frequency (MHz)	(600.1929, 600.1929)
Nucleus	(1H, 1H)
Number of Transients	8
Origin	spect
Original Points Count	(1024, 256)
Owner	nmr
Points Count	(1024, 1024)
Pulse Sequence	noesygpphpp
Solvent	DMSO-d6
Spectrum Type	NOESY
Sweep Width (Hz)	(5741.51, 5741.51)
Temperature (degree C)	23.993
Title	

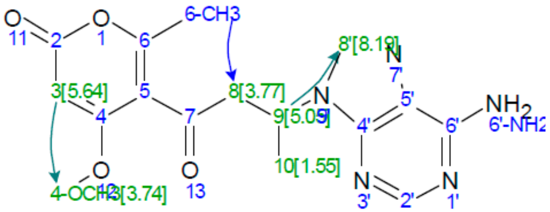

Figure S 9. NOESY of compound **1**

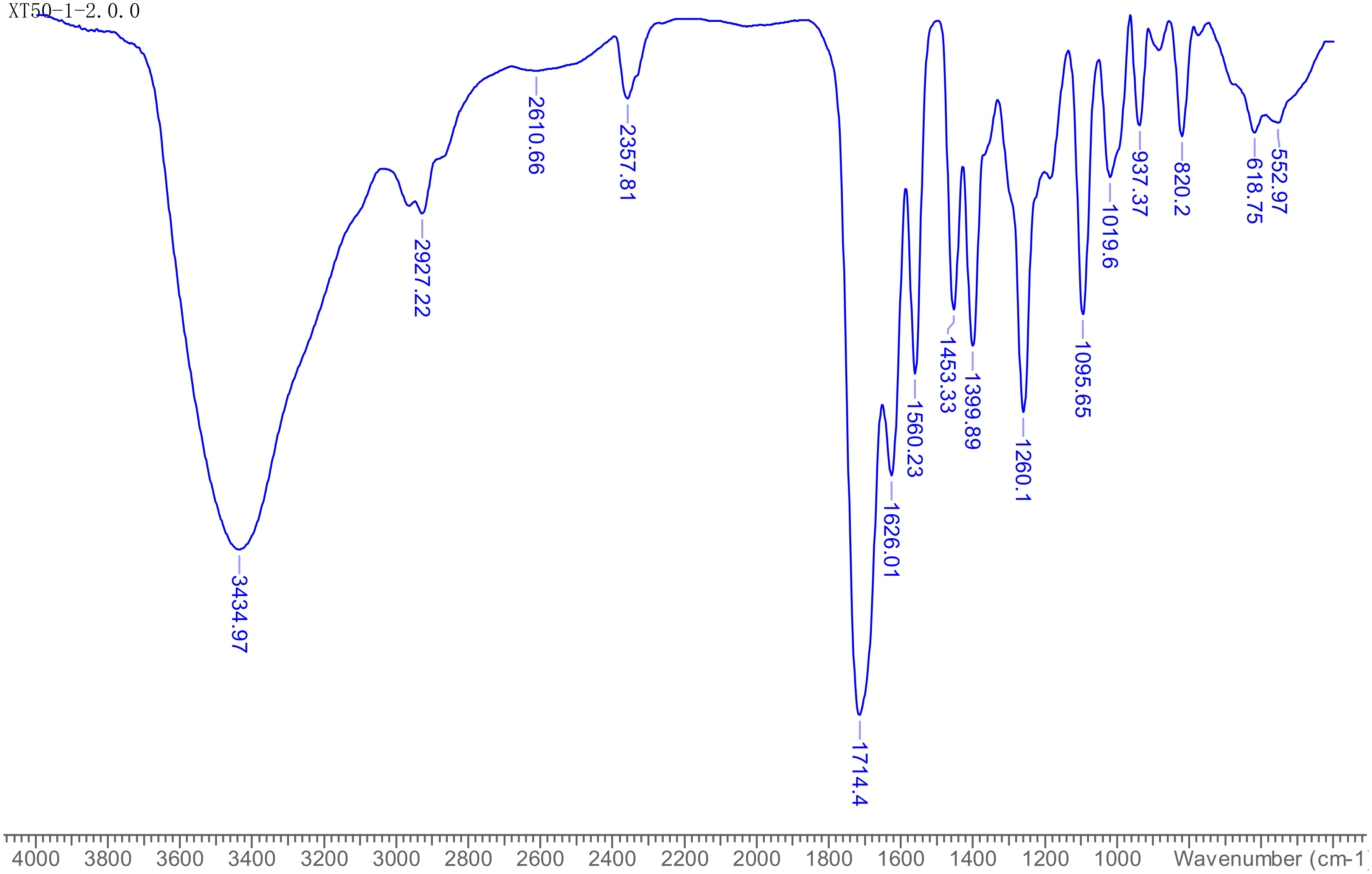

Figure S 10. IR spectrum of compound **2**

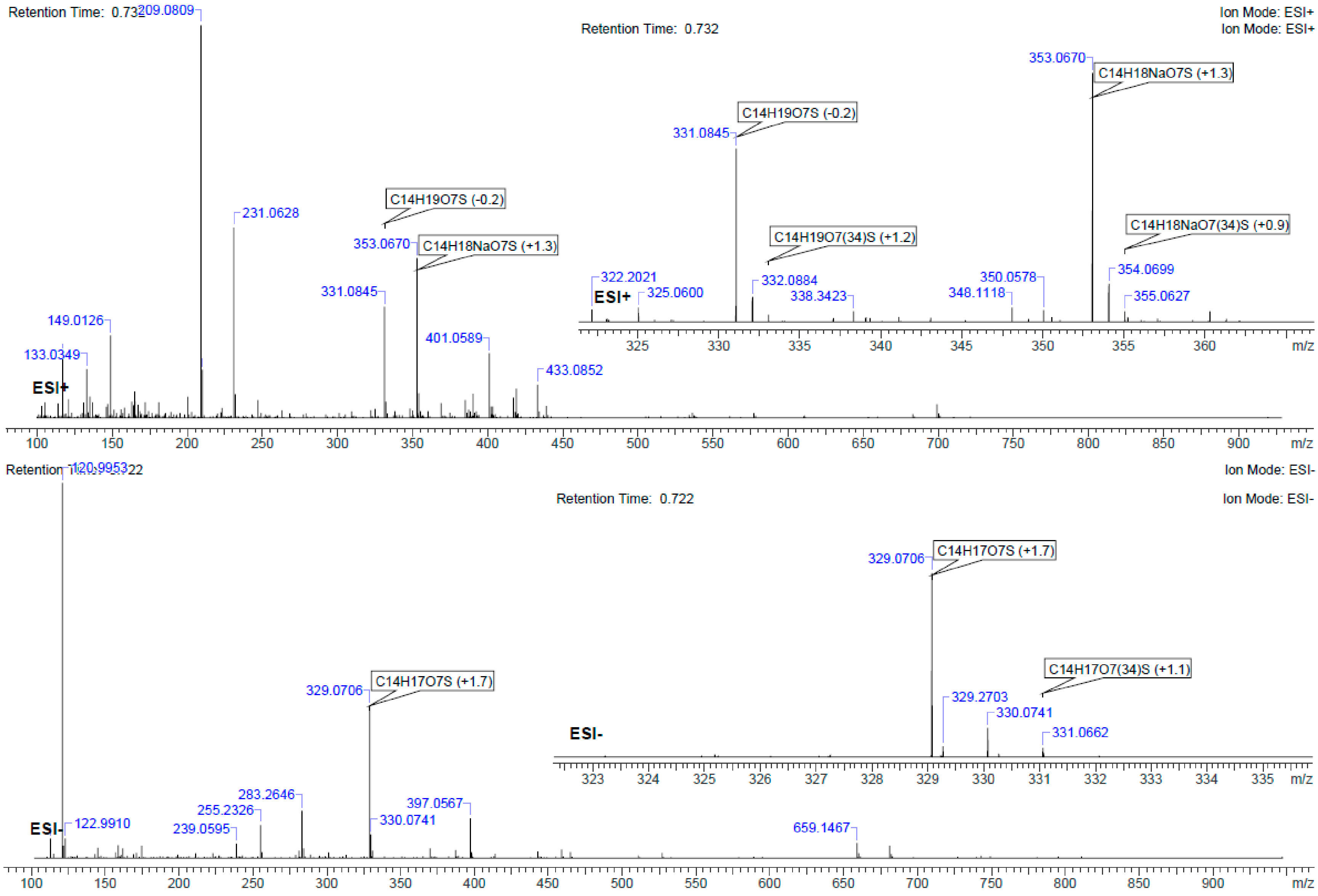

Figure S 11. HR-ESI-MS spectra of compound **2**

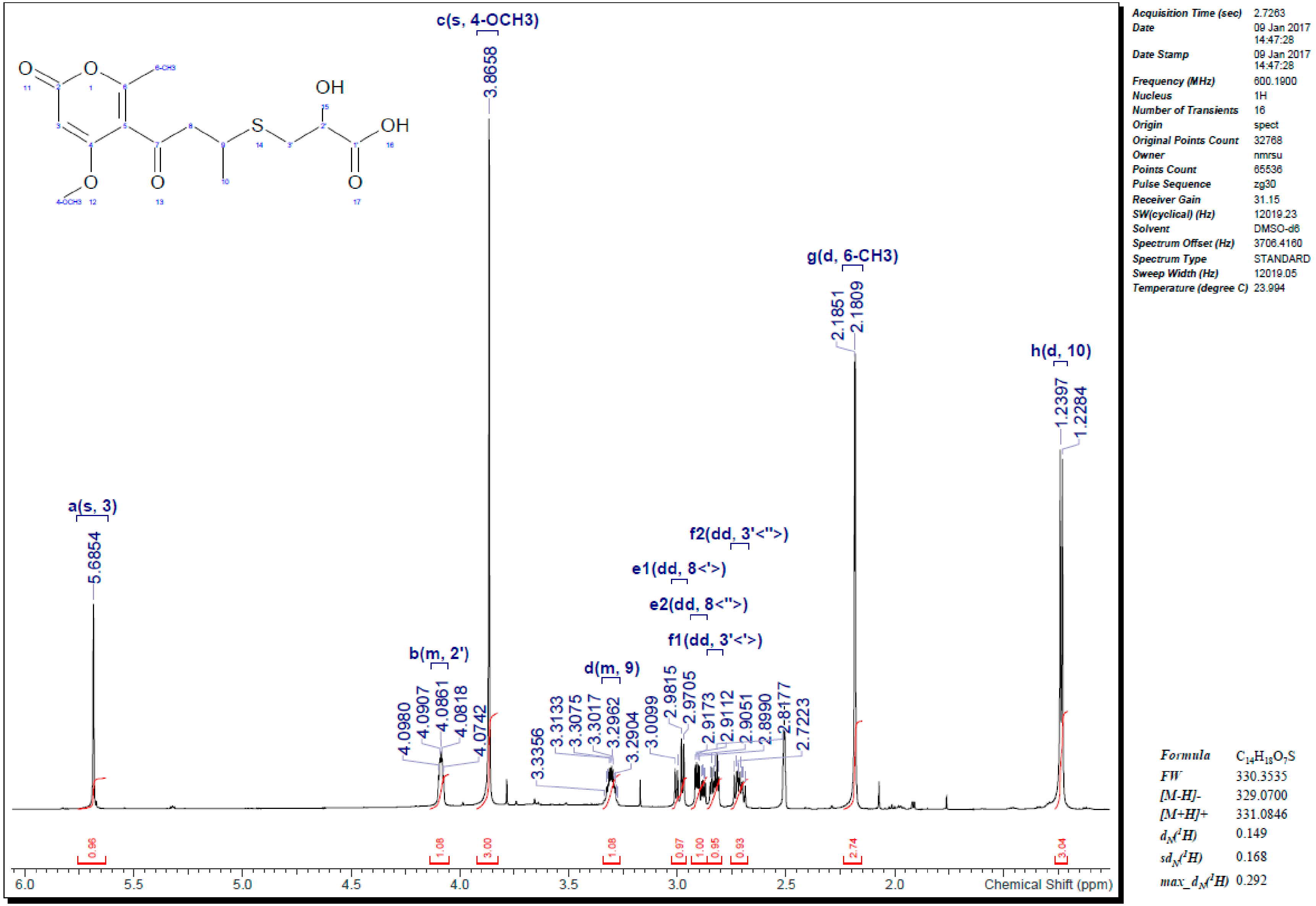

Figure S 12. ^{1}H-NMR (600 MHz, DMSO-d_6) of compound **2**

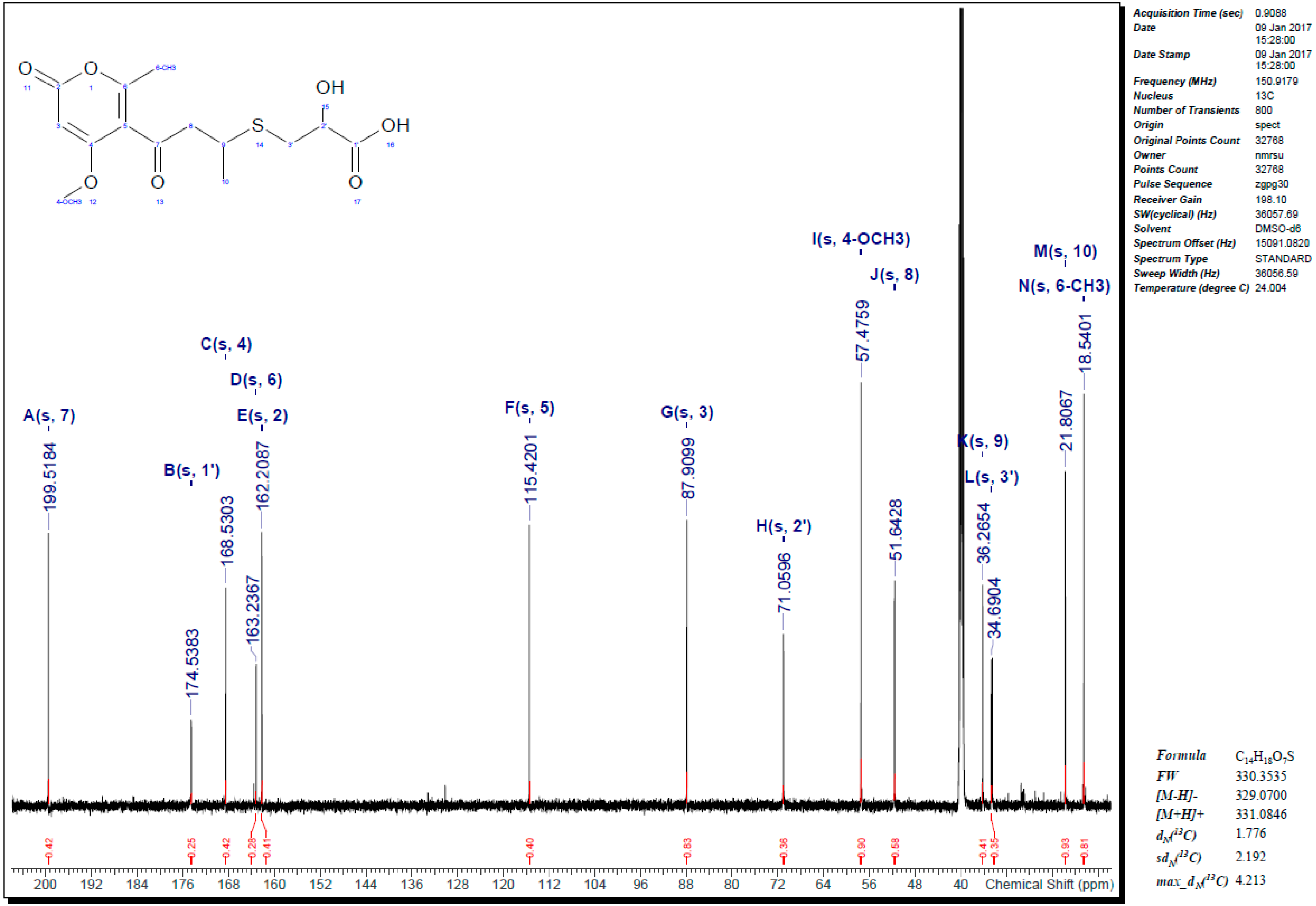

Figure S 13. ^{13}C-NMR (150 MHz, DMSO-d_6) of compound **2**

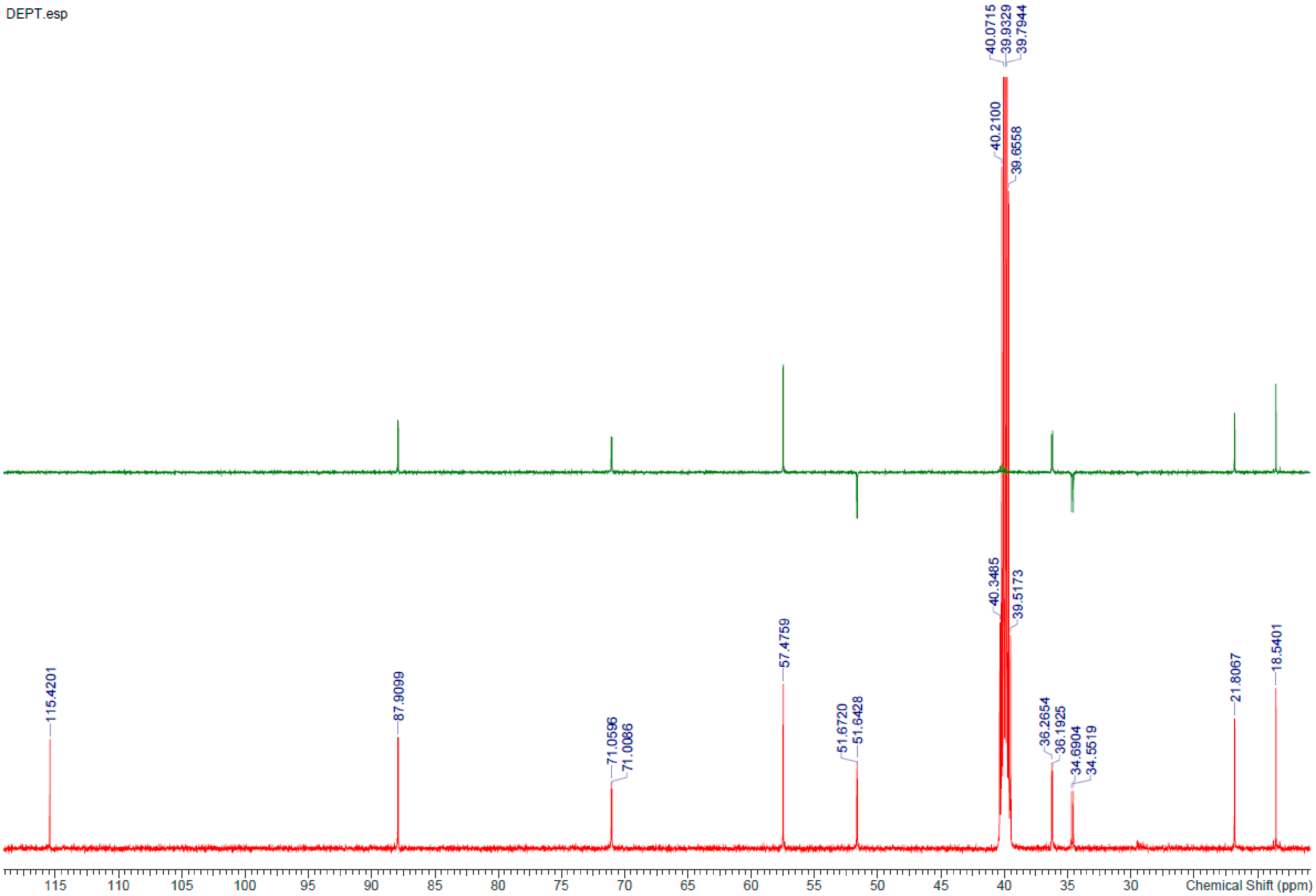

Figure S 14. DEPT of compound **2**

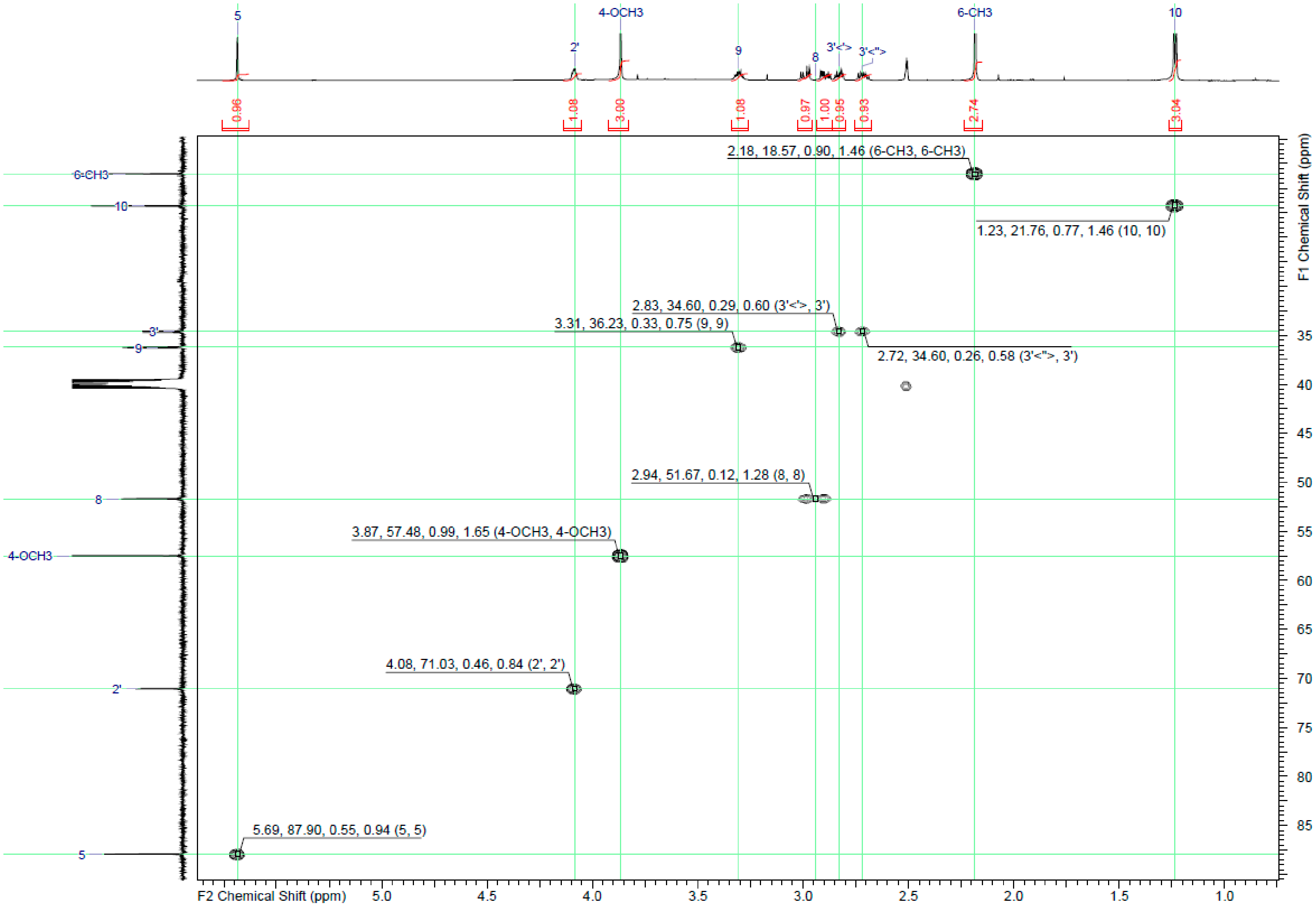

Figure S 15. HSQC of compound **2**

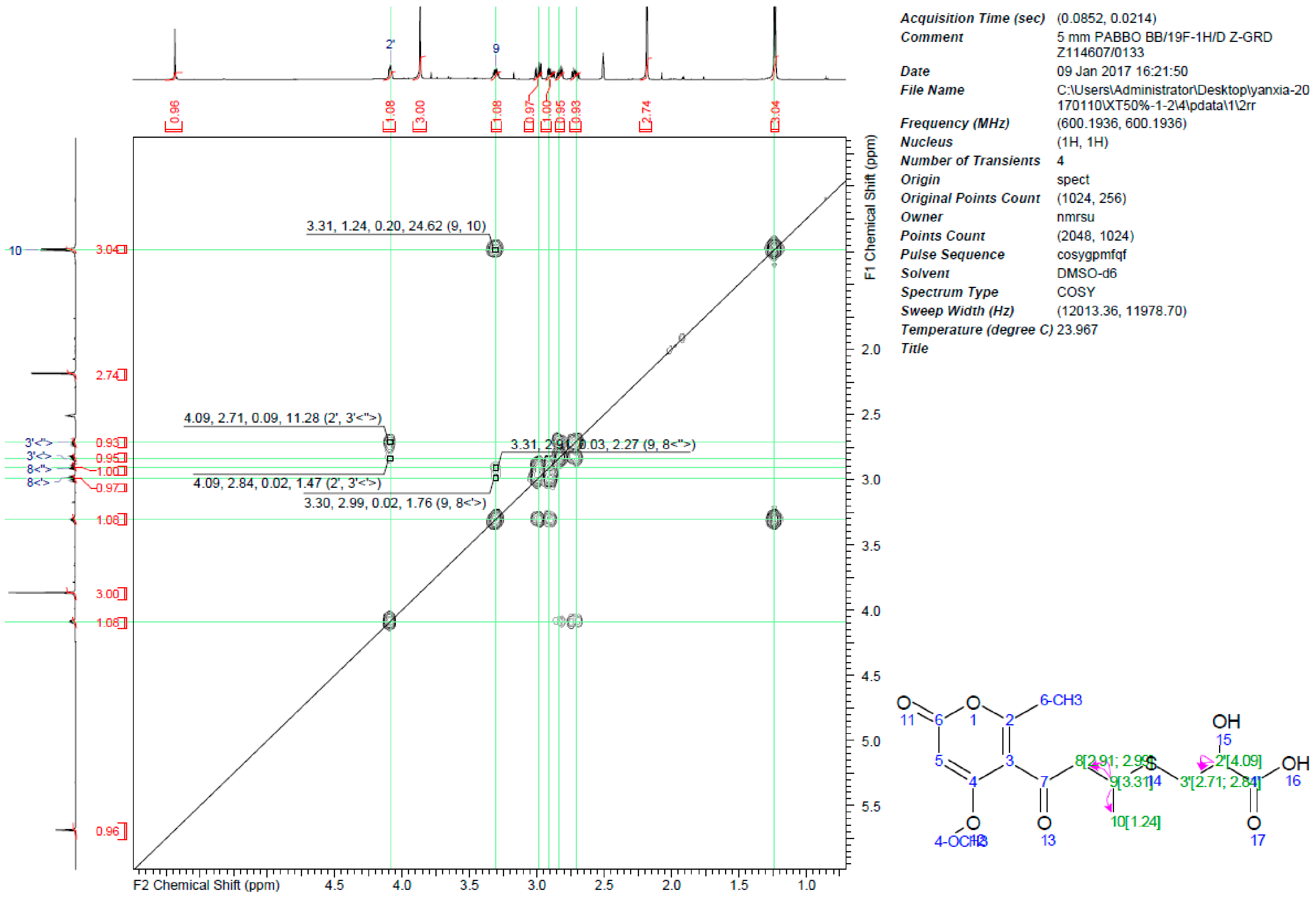

Figure S 16. ^1H-^1H COSY of compound **2**

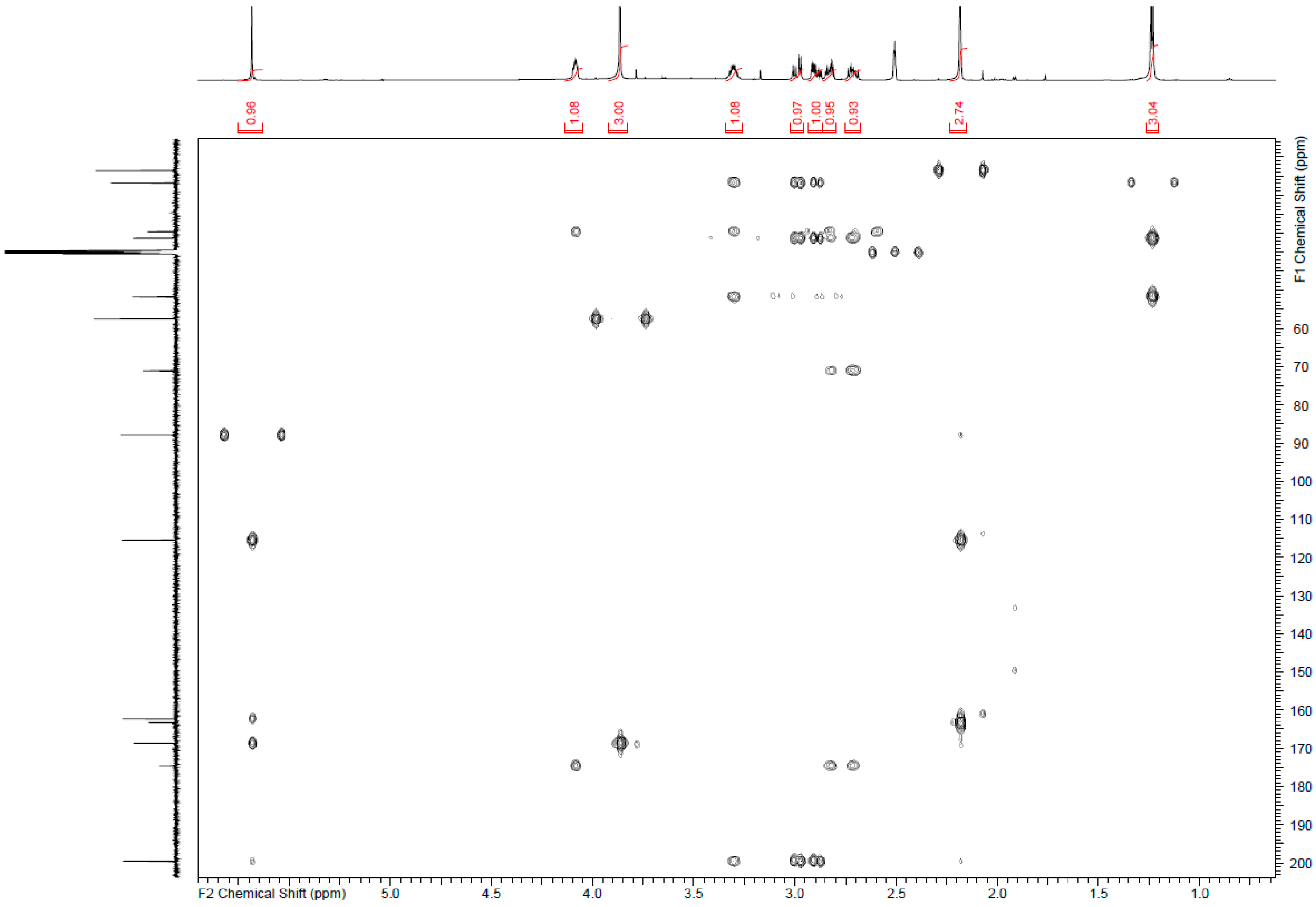

Figure S 17. HMBC of compound **2**

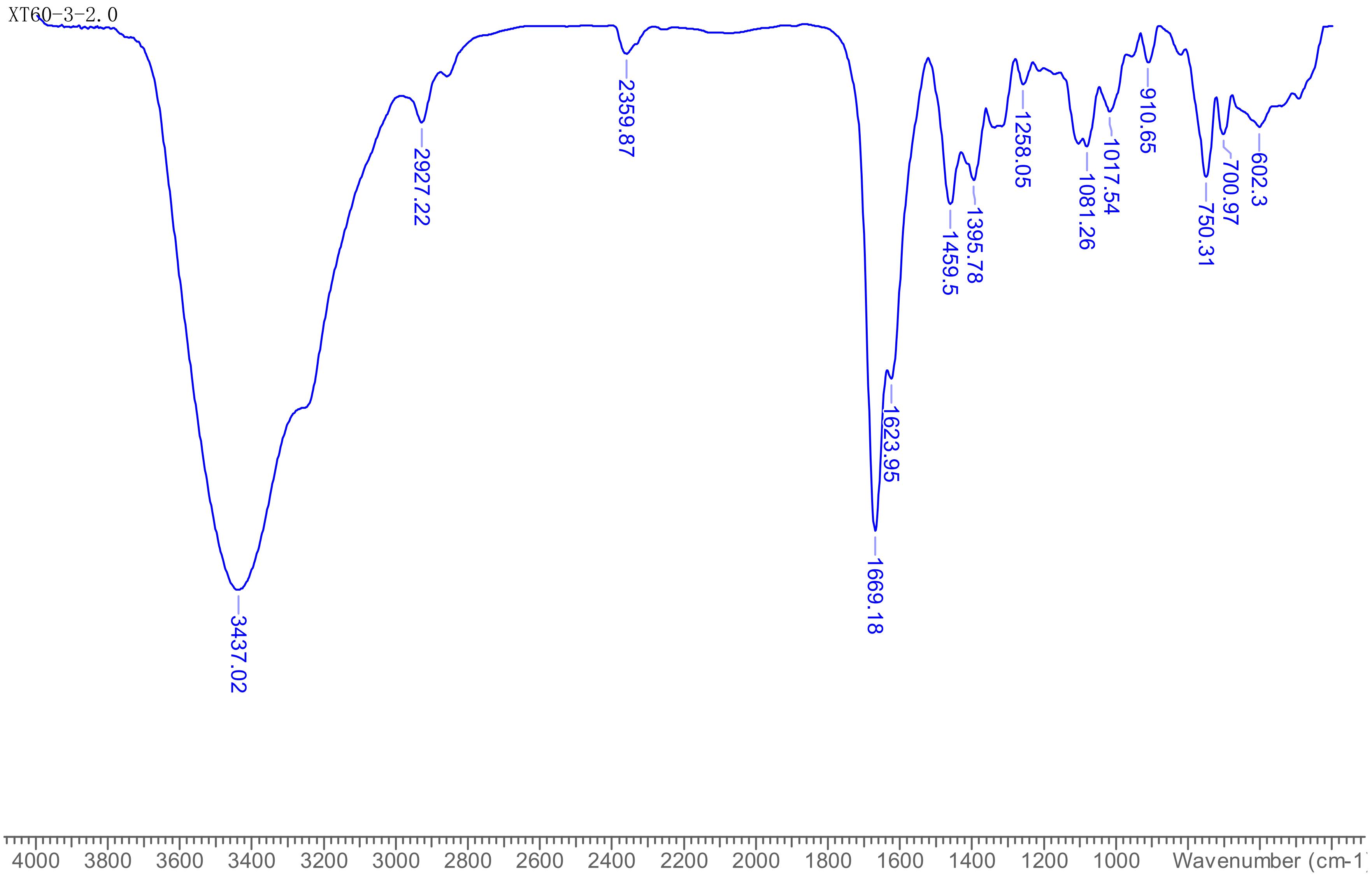

Figure S 18. IR spectrum of compound **3**

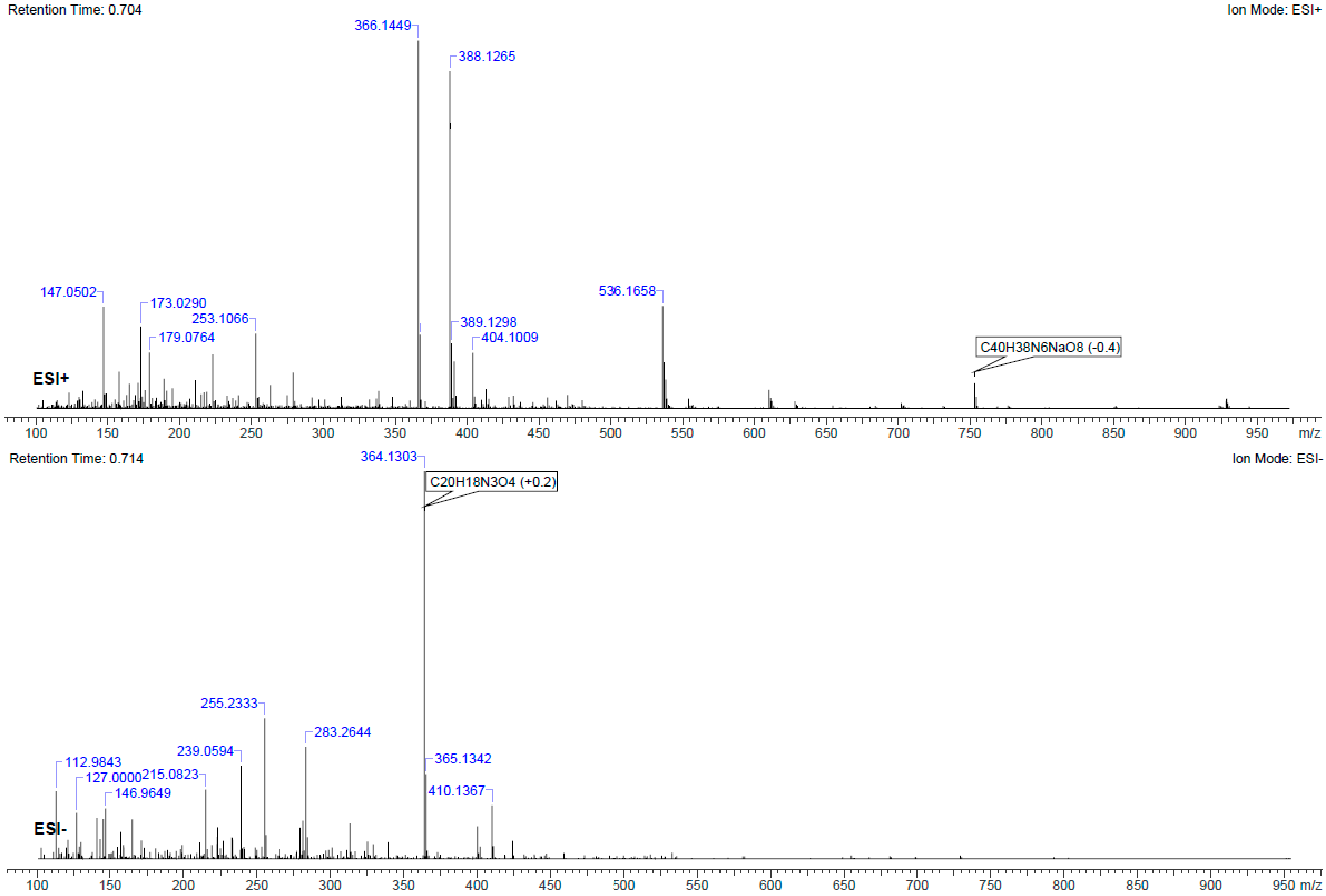

Figure S 19. HR-ESI-MS spectra of compound **3**

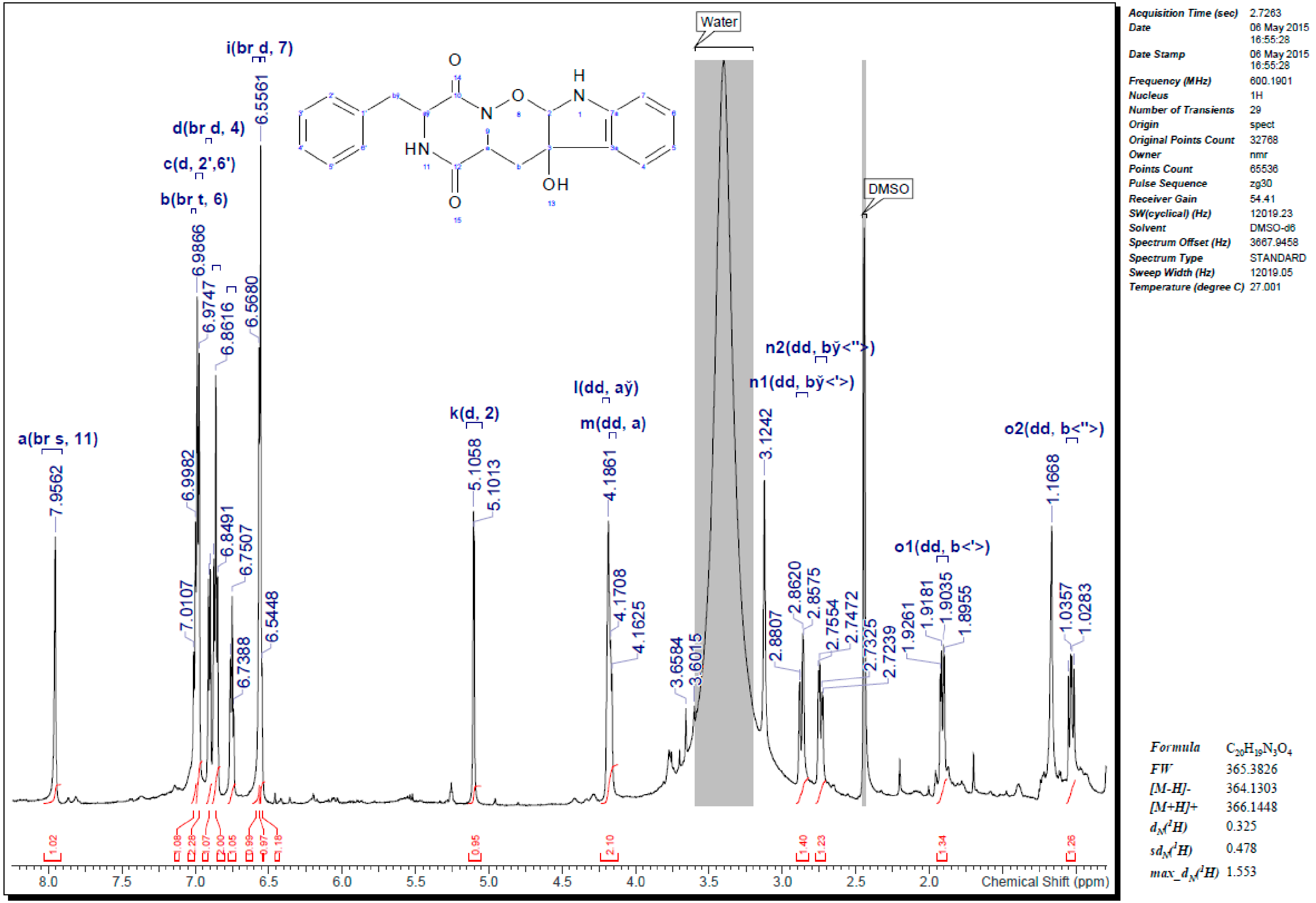

Figure S 20. ¹H-NMR (600 MHz, DMSO-d_6) of compound **3**

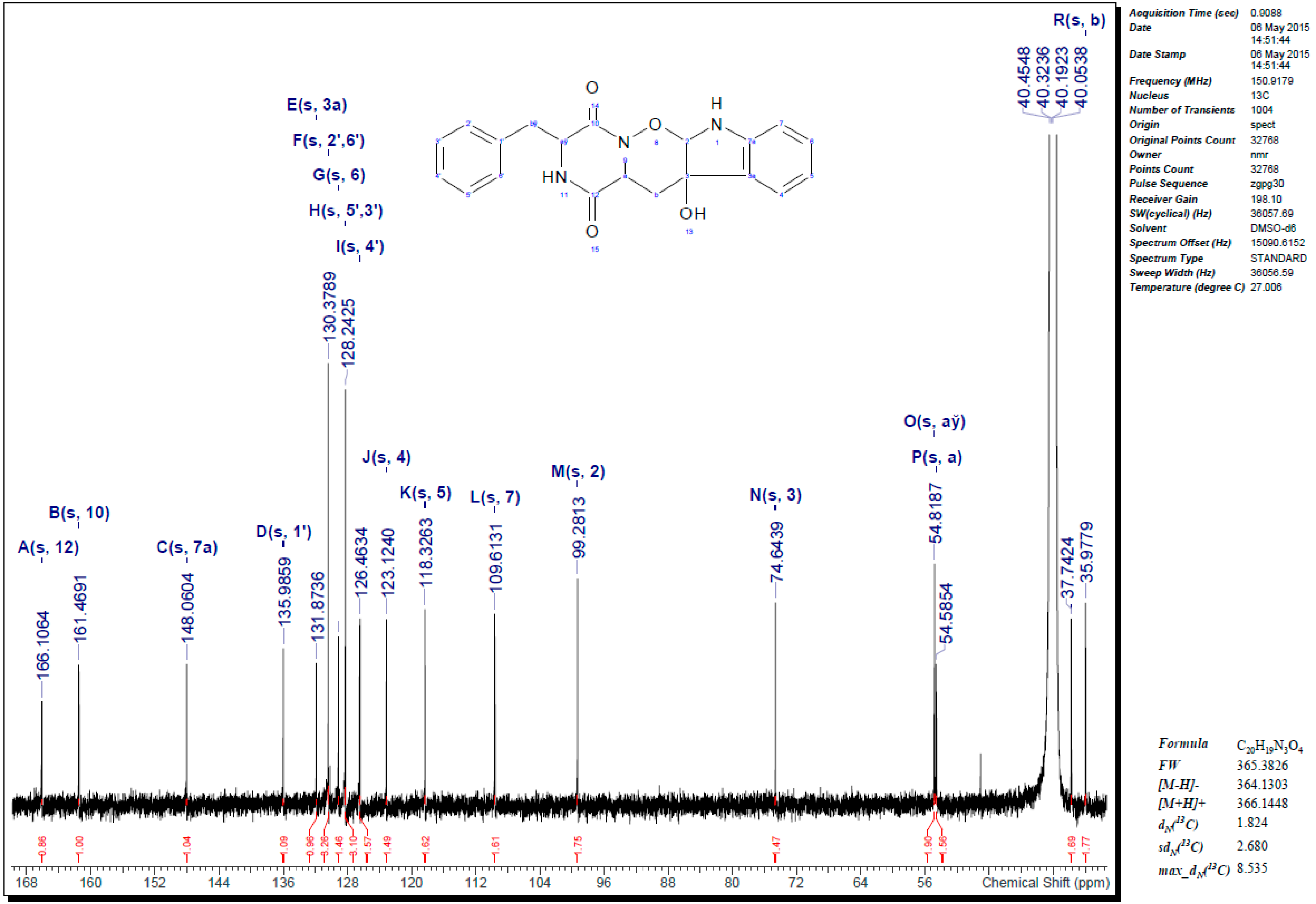

Figure S 21. ^{13}C-NMR (150 MHz, DMSO-d_6) of compound **3**

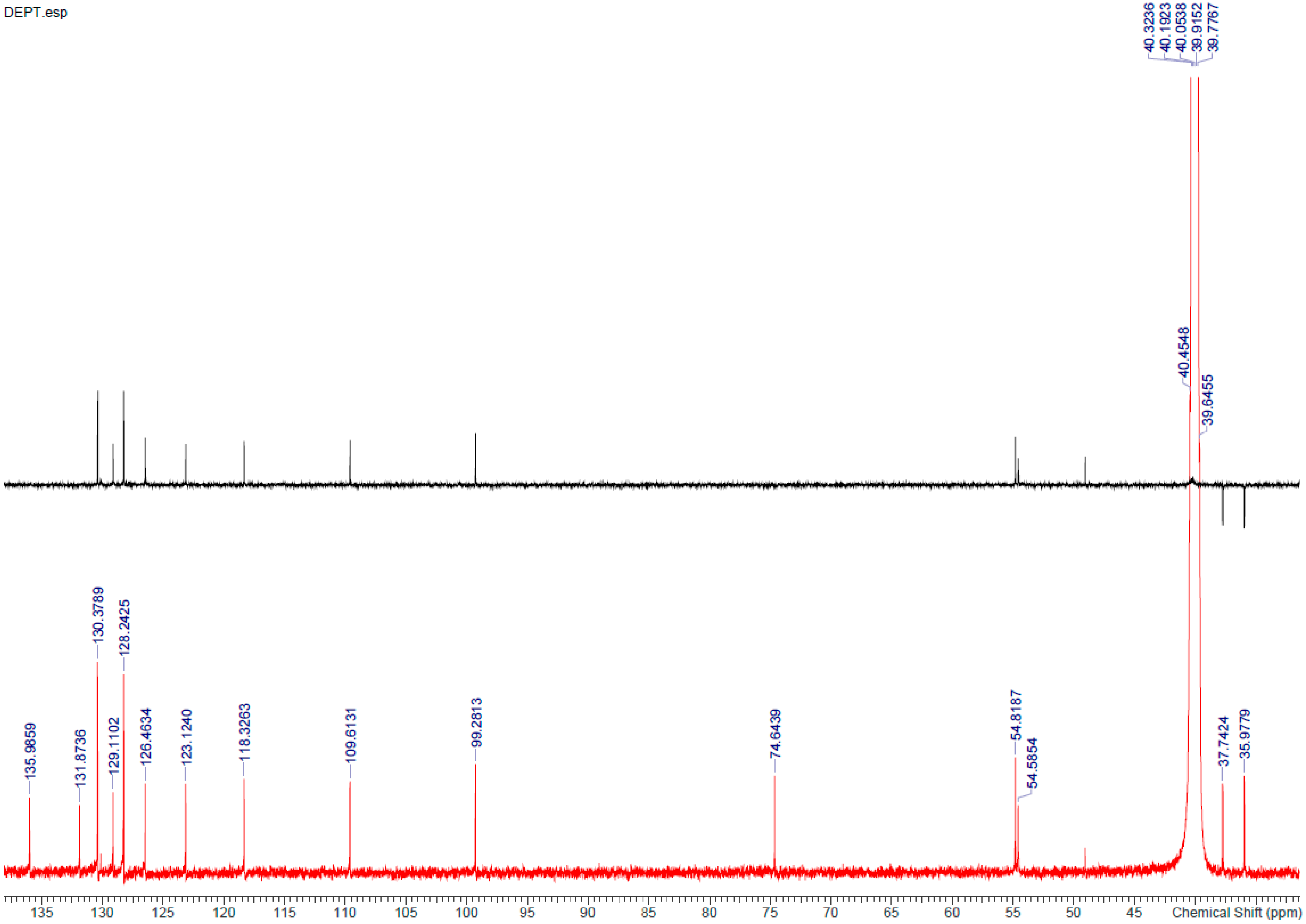

Figure S 22. DEPT of compound **3**

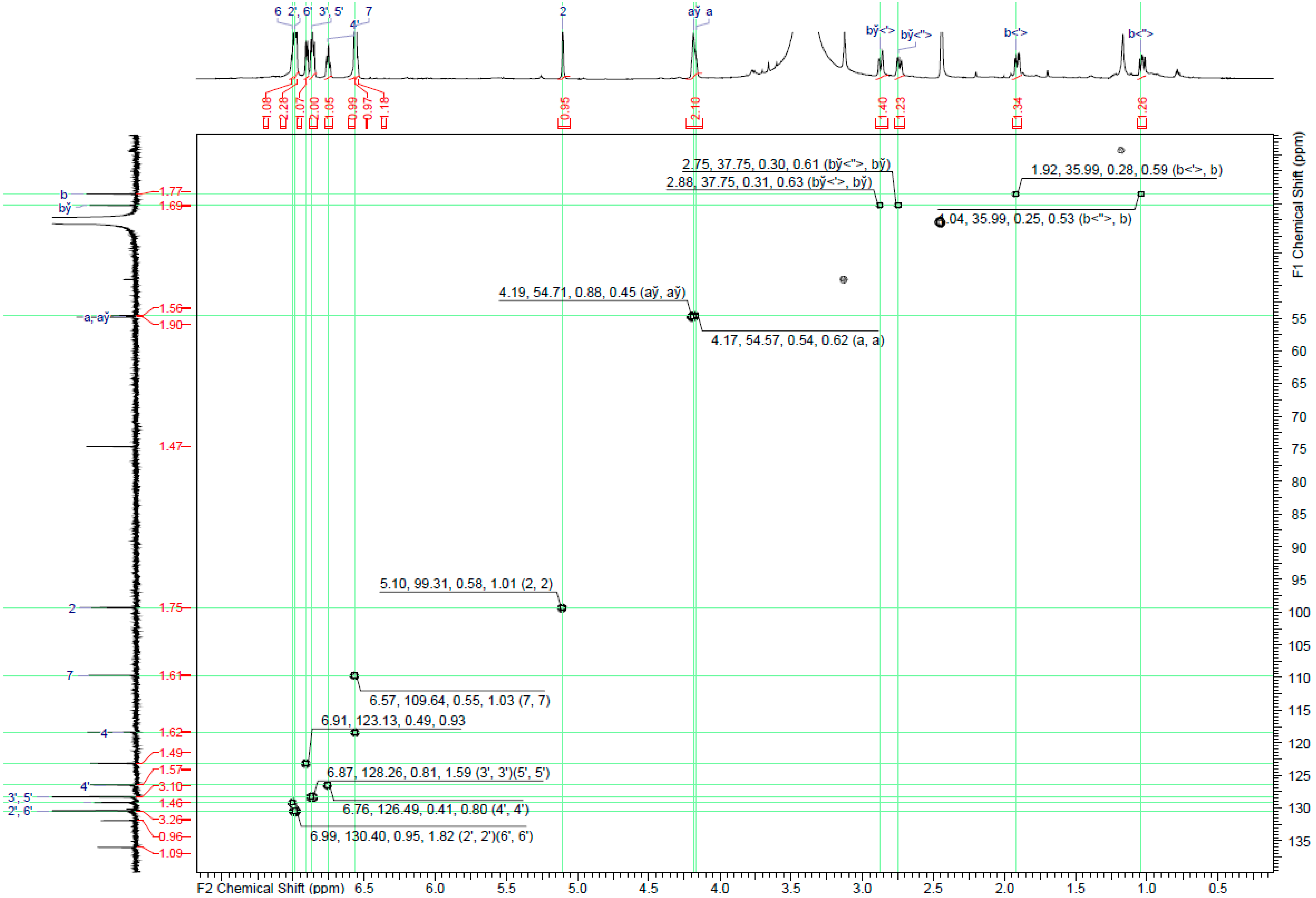

Figure S 23. HSQC of compound **3**

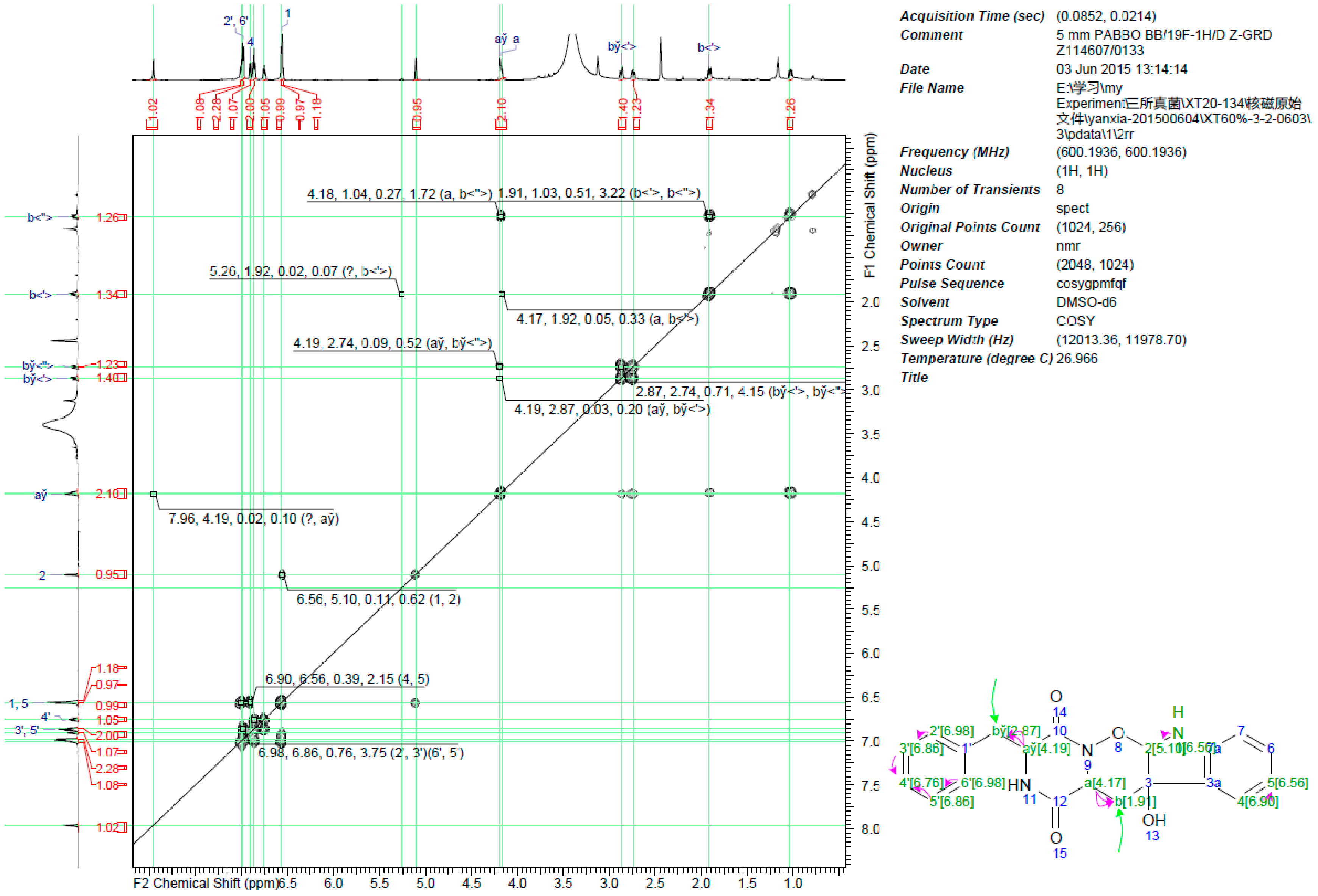

Figure S 24. ^{1}H-^{1}H COSY of compound **3**

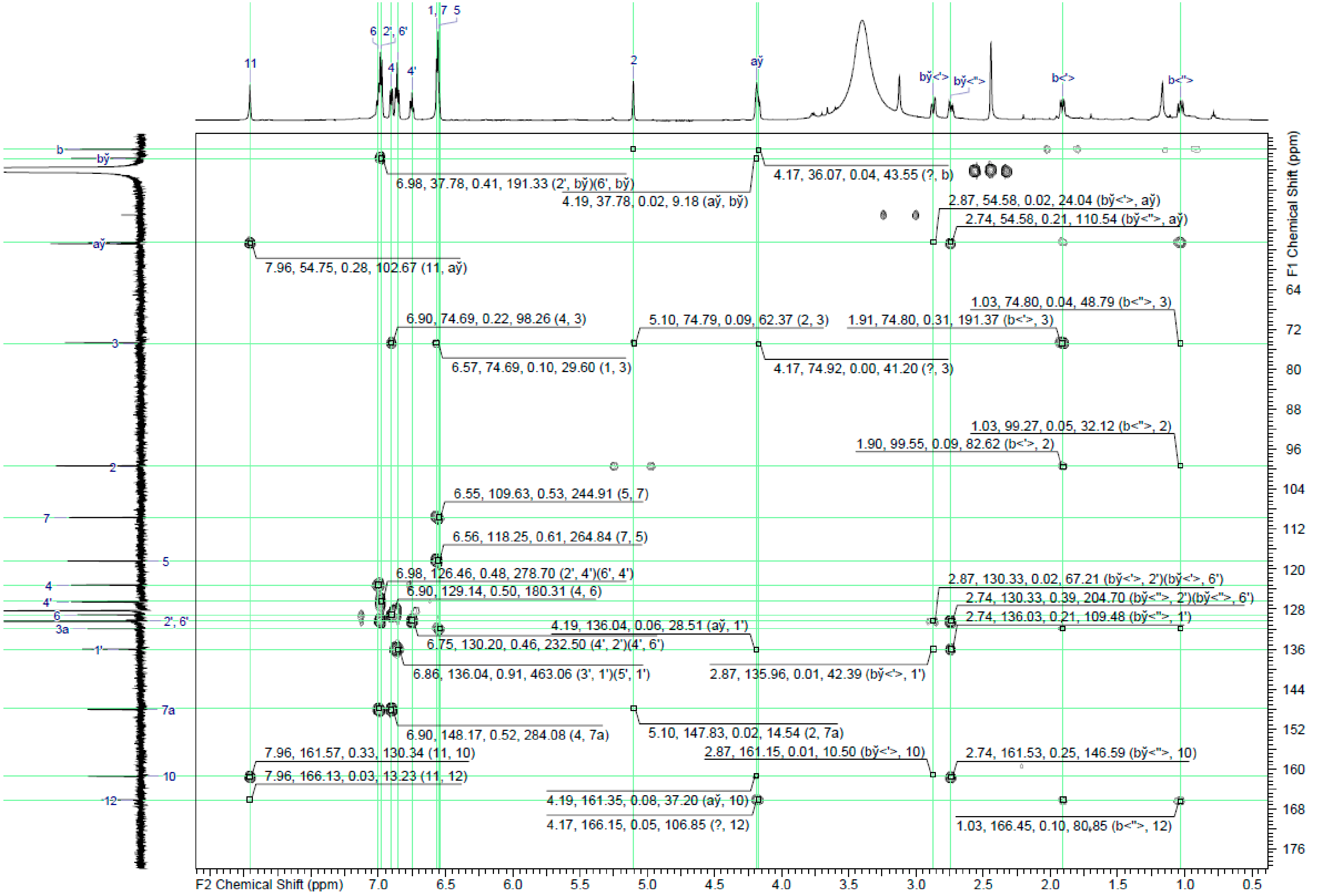

Figure S 25. HMBC of compound **3**

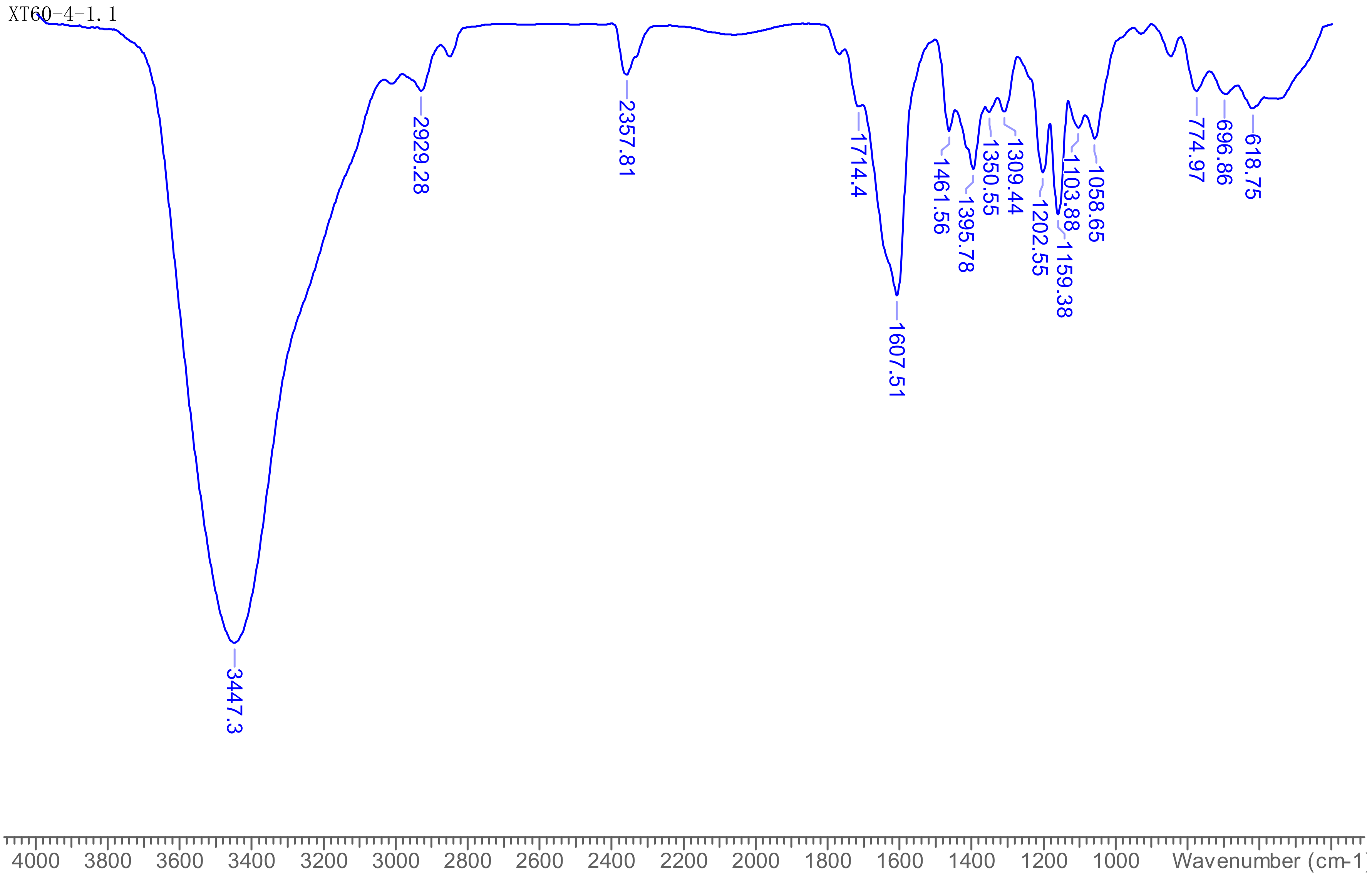

Figure S 26. IR spectrum of compound **4**

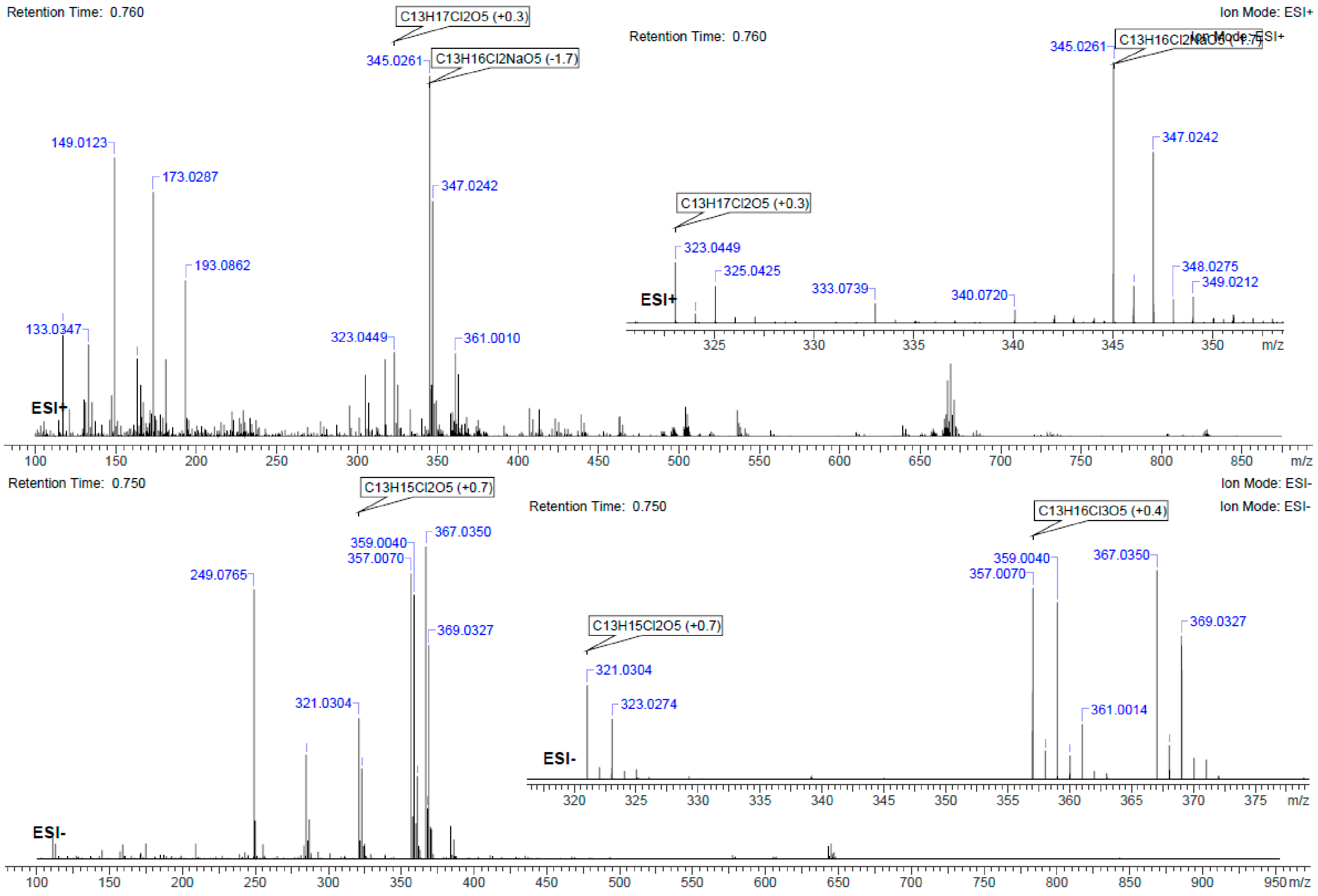

Figure S 27. HR-ESI-MS spectra of compound **4**

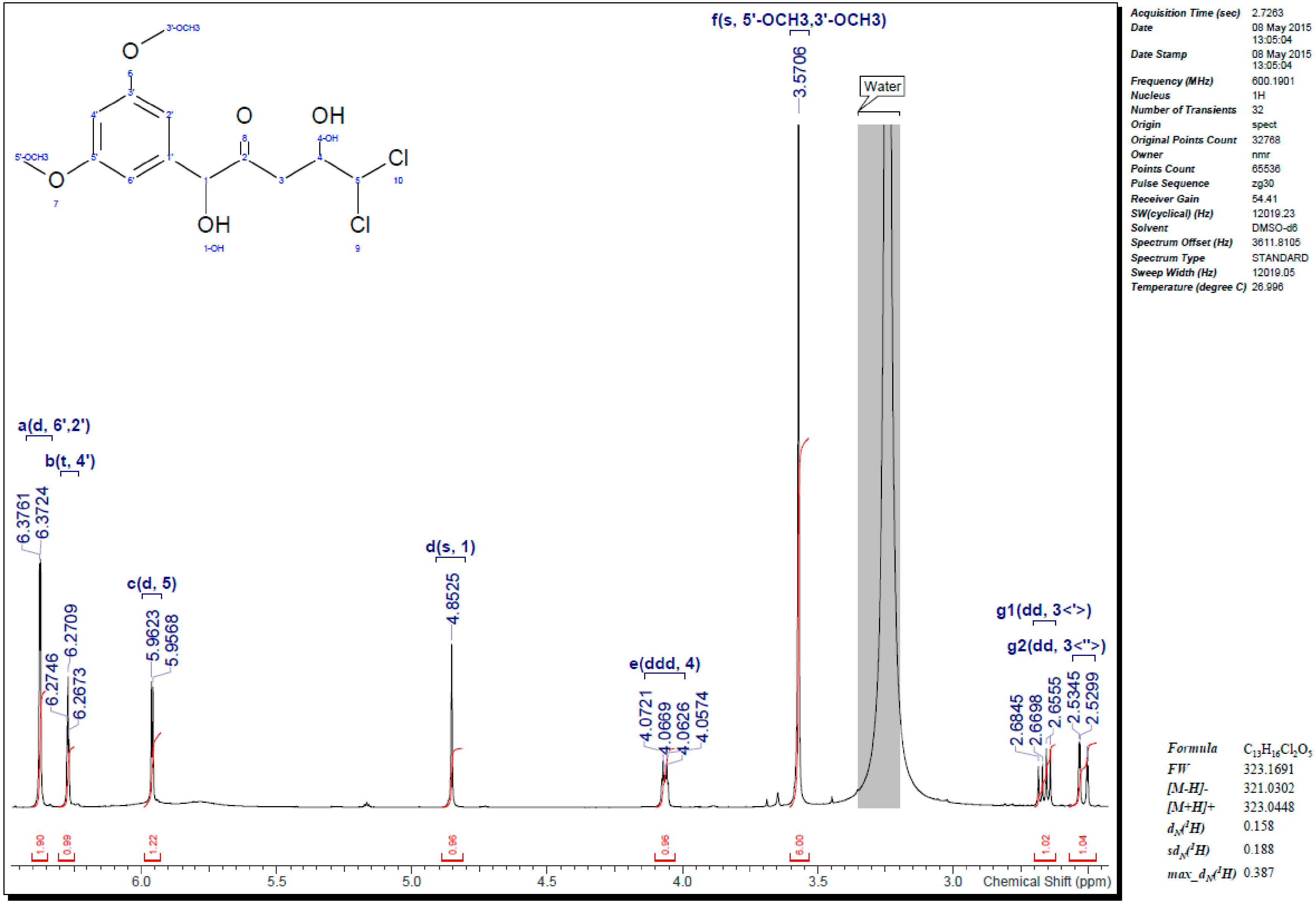

Figure S 28. ^{1}H-NMR (600 MHz, DMSO-d_6) of compound **4**

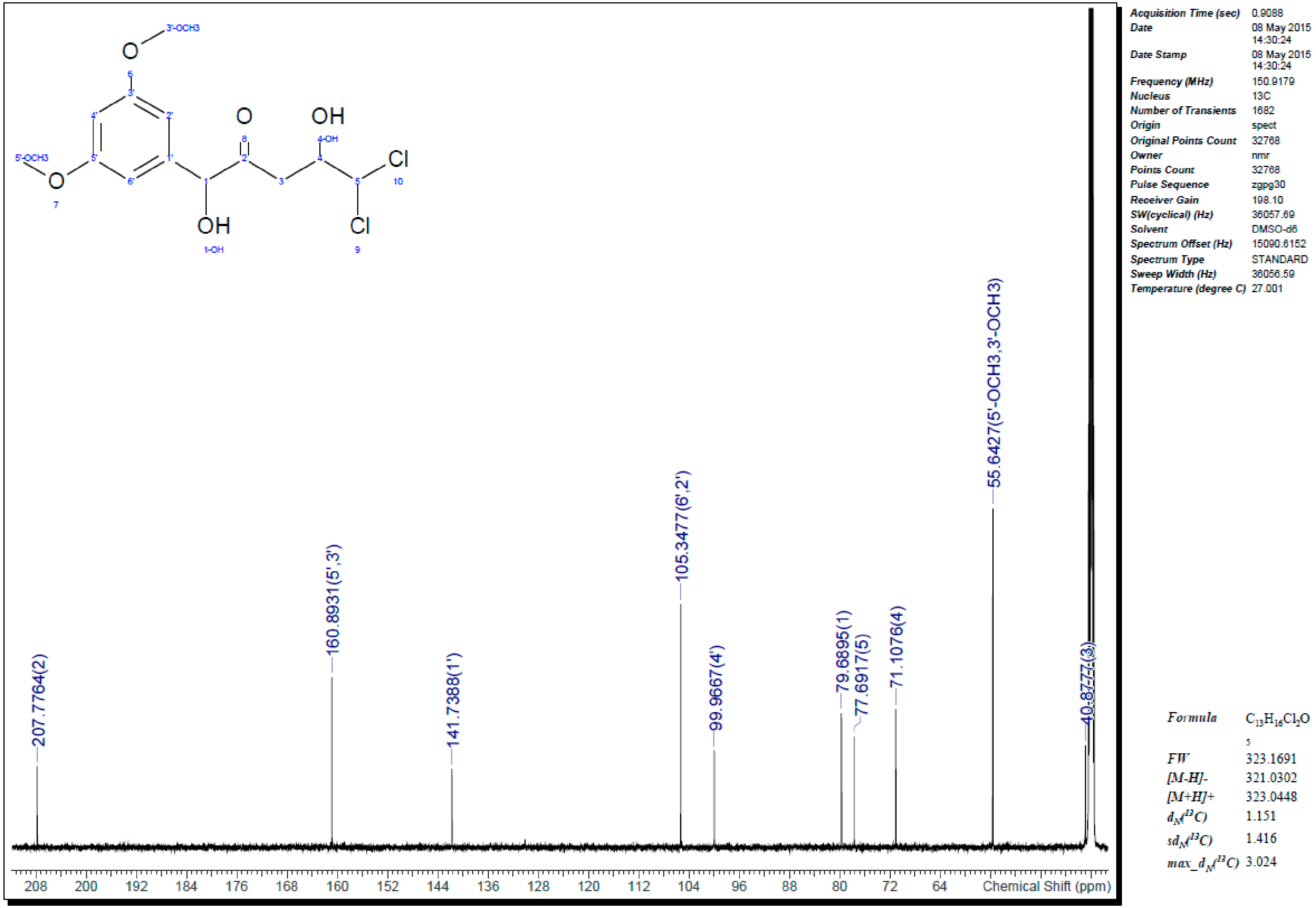

Figure S 29. ^{13}C-NMR (150 MHz, DMSO-d_6) of compound **4**

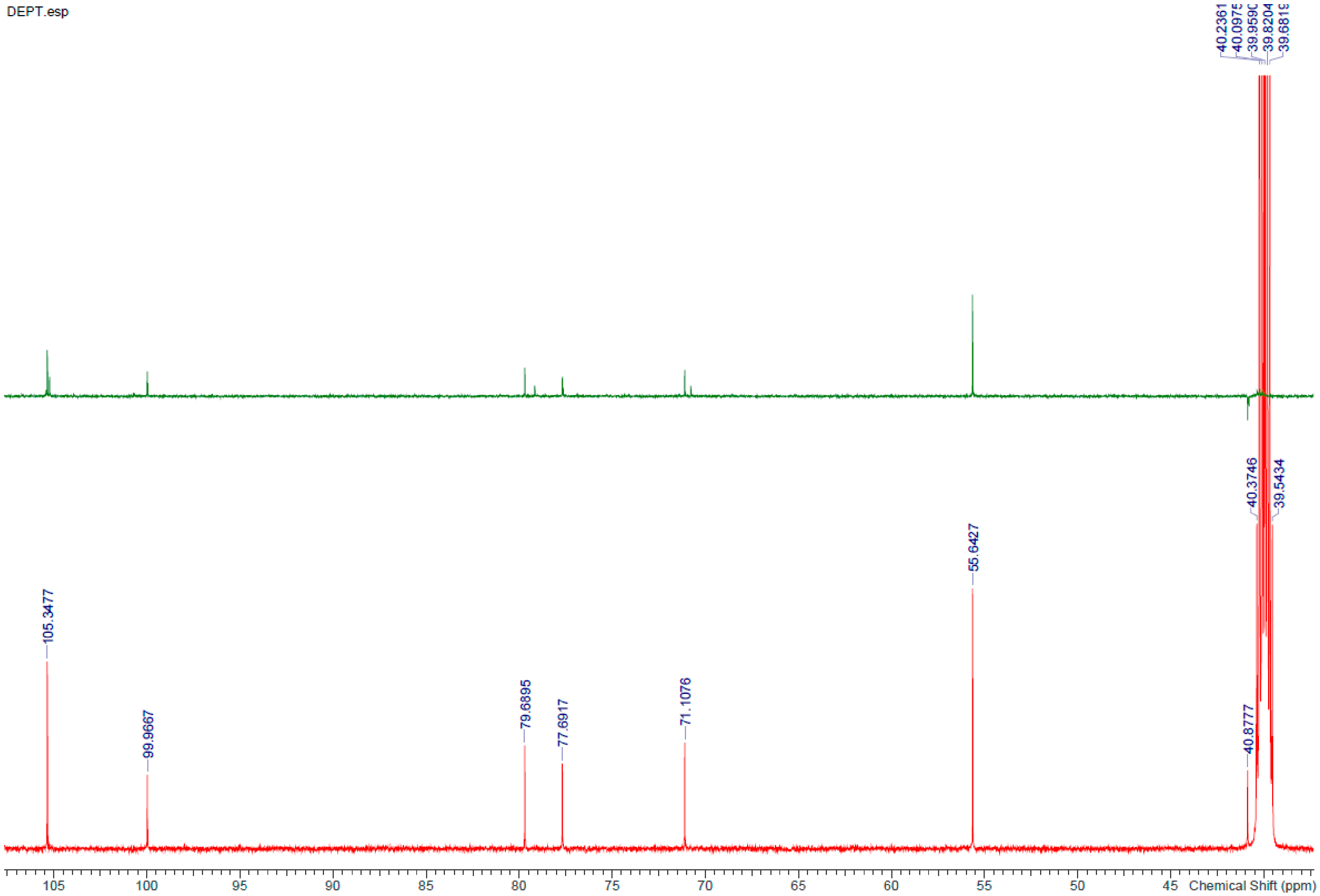

Figure S 30. DEPT of compound **4**

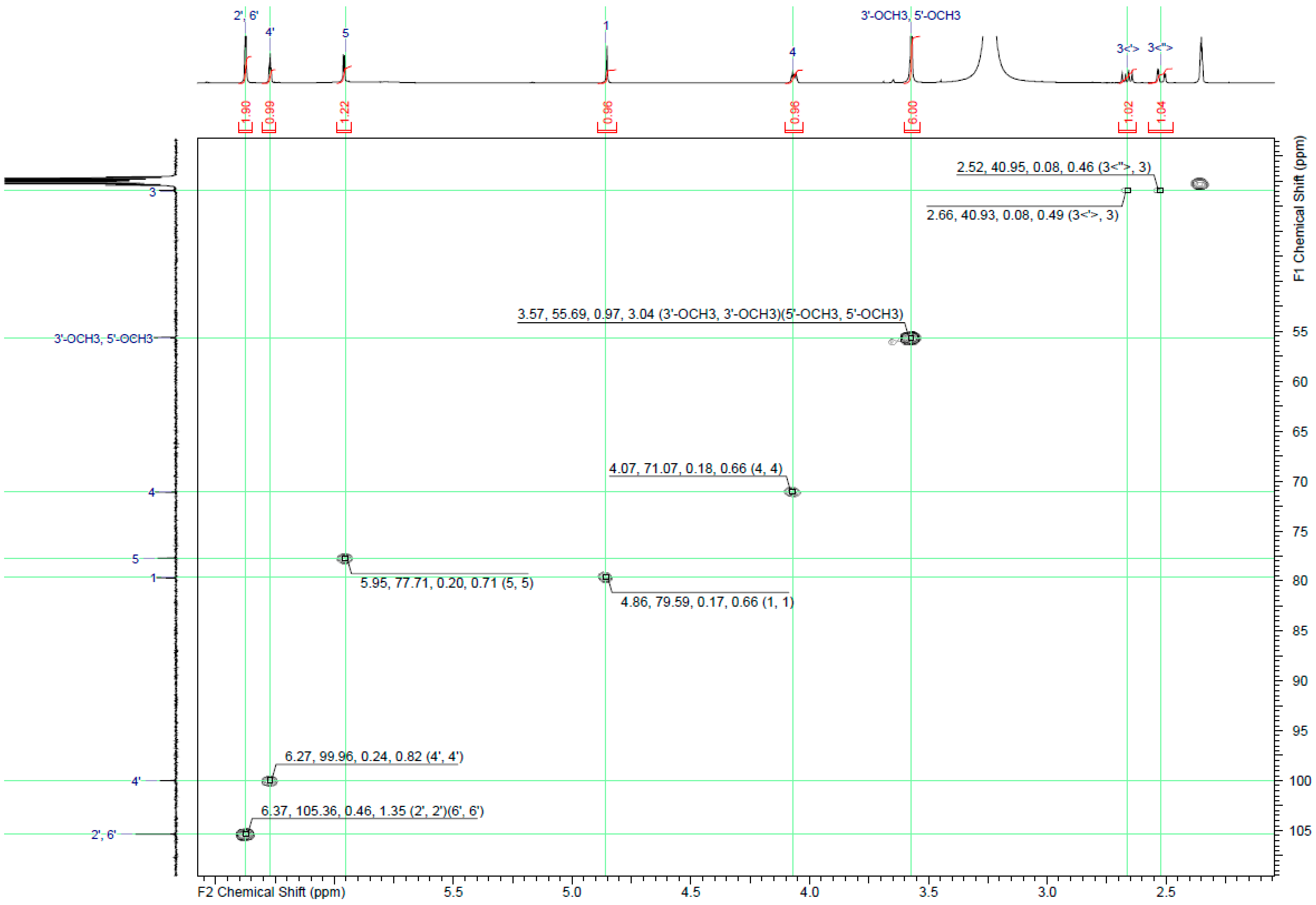

Figure S 31. HSQC of compound **4**

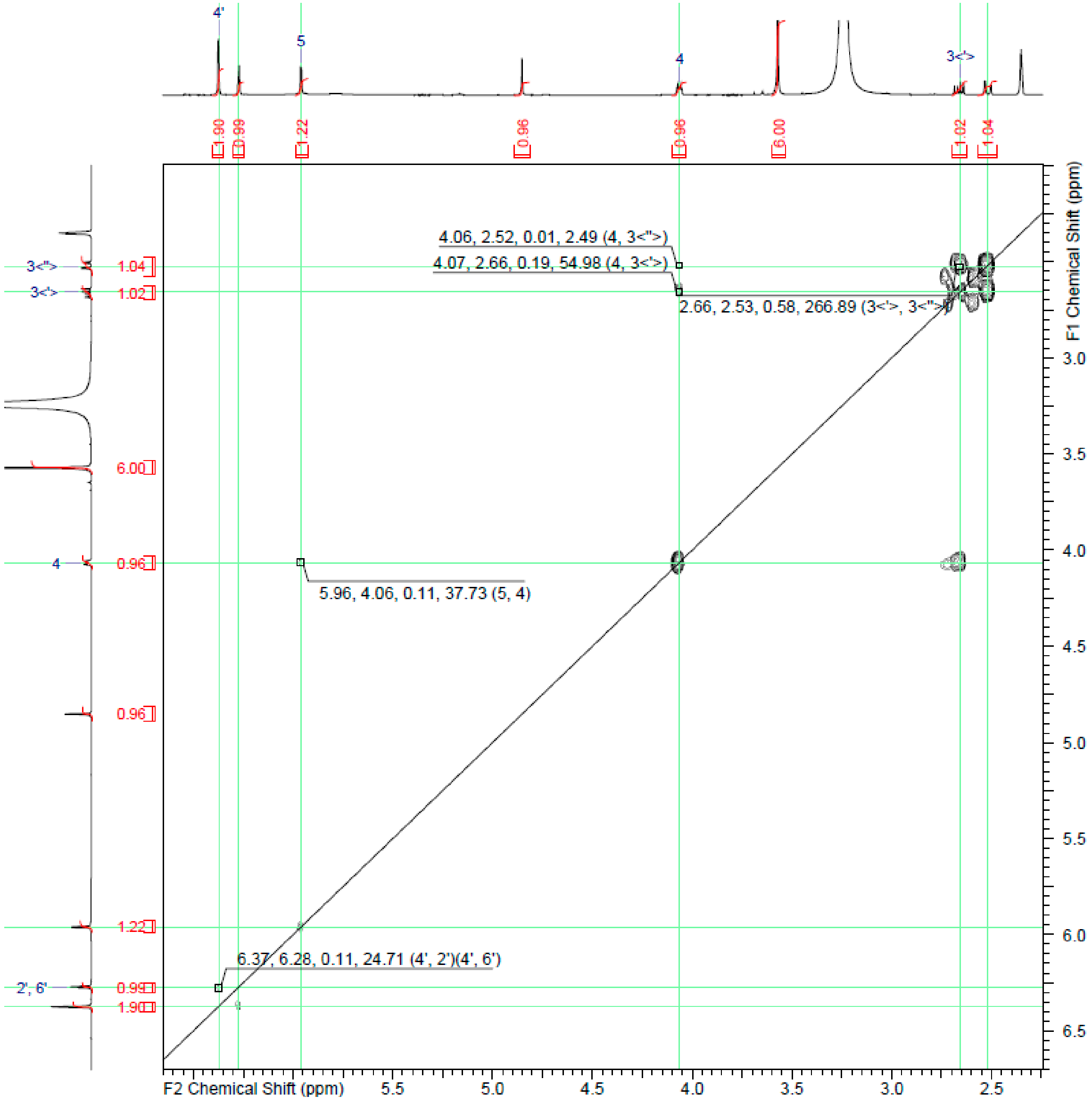

Acquisition Time (sec)	(0.0852, 0.0214)
Comment	5 mm PABBO BB/19F-1H/D Z-GRD Z114607/0133
Date	18 Jun 2015 16:39:18
File Name	C:\Users\Administrator\Desktop\YANXIA-20150619\XT60%-4-1-0618\3\pdata\1\2rr
Frequency (MHz)	(600.1936, 600.1936)
Nucleus	(1H, 1H)
Number of Transients	8
Origin	spect
Original Points Count	(1024, 256)
Owner	nmr
Points Count	(2048, 1024)
Pulse Sequence	cosygpmfqf
Solvent	DMSO-d6
Spectrum Type	COSY
Sweep Width (Hz)	(12013.36, 11978.70)
Temperature (degree C)	26.962
Title	

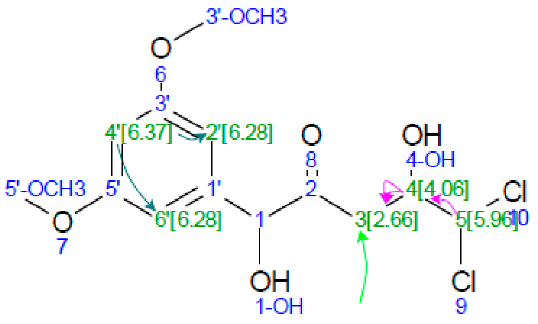

Figure S 32. ^1H-^1H COSY of compound **4**

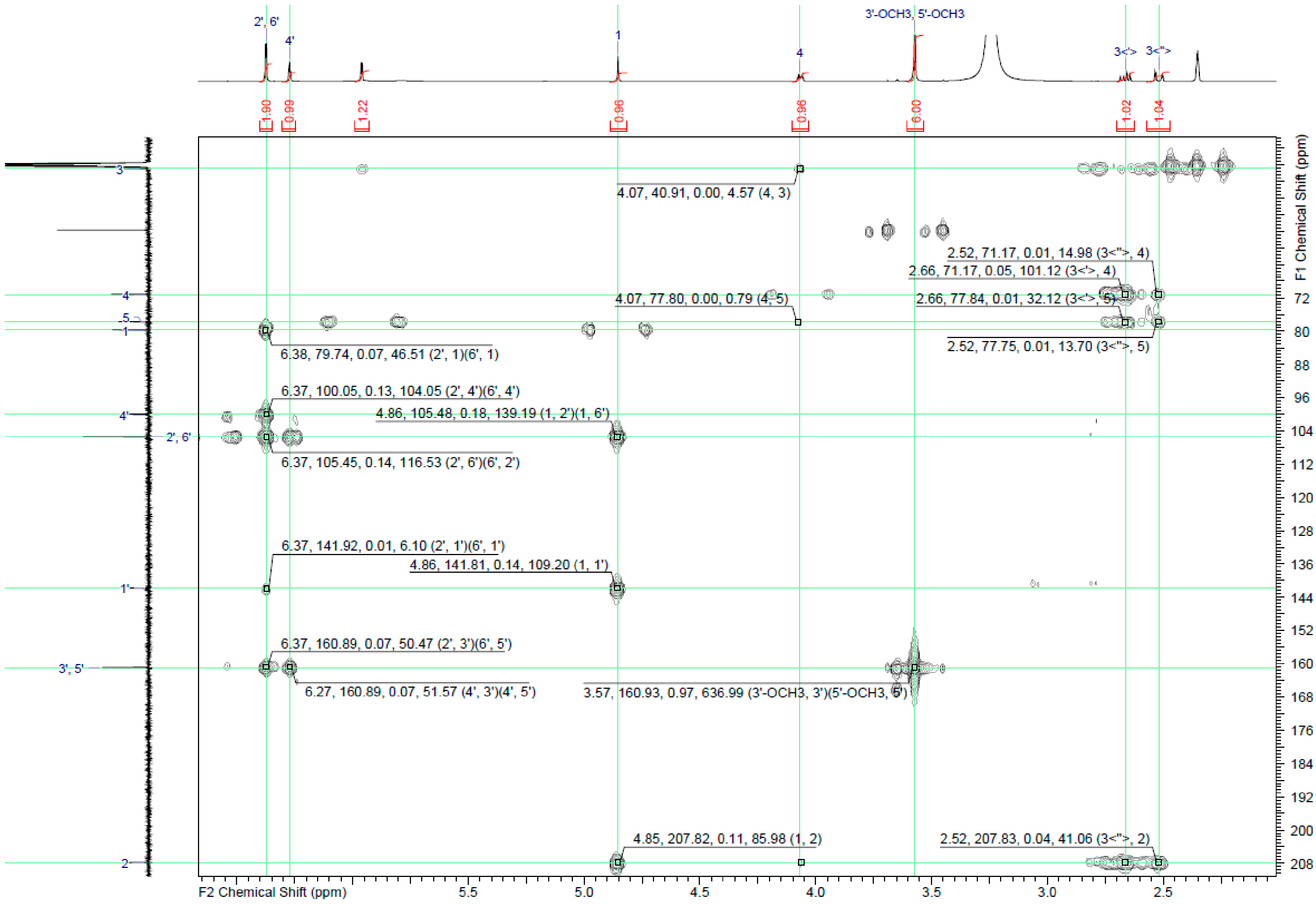

Figure S 33. HMBC of compound **4**

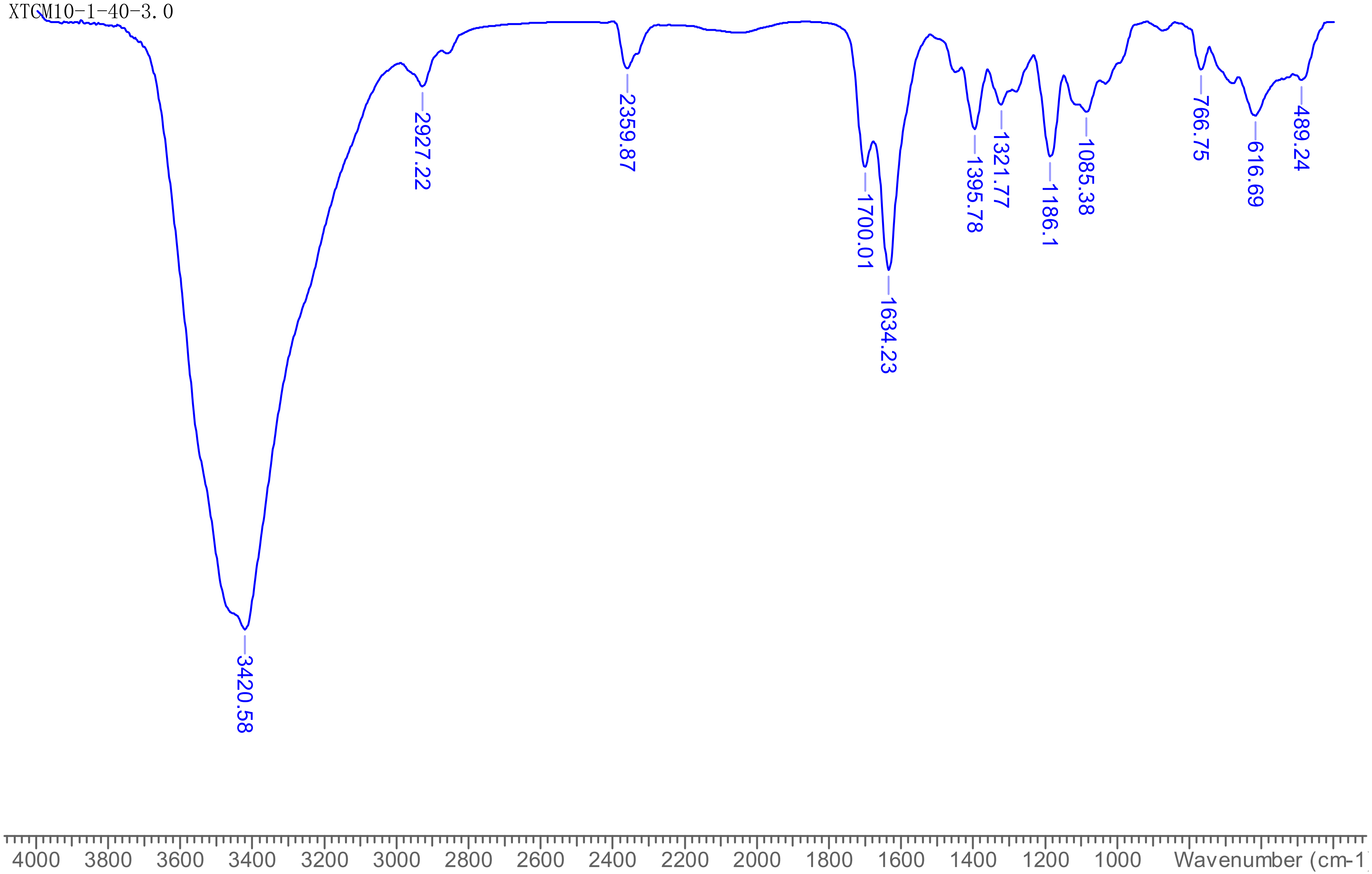

Figure S 34. IR spectrum of compound **5**

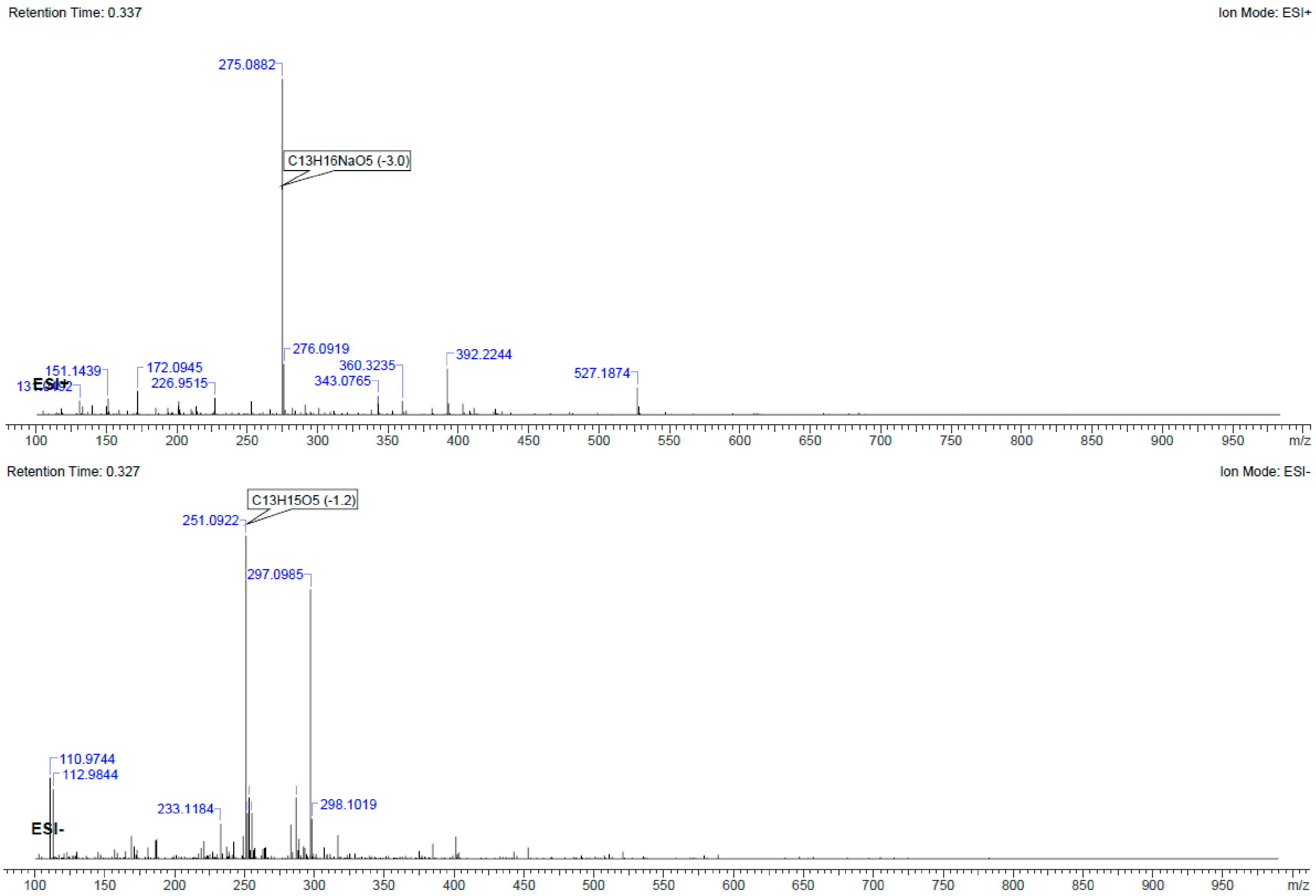

Figure S 35. HR-ESI-MS spectra of compound **5**

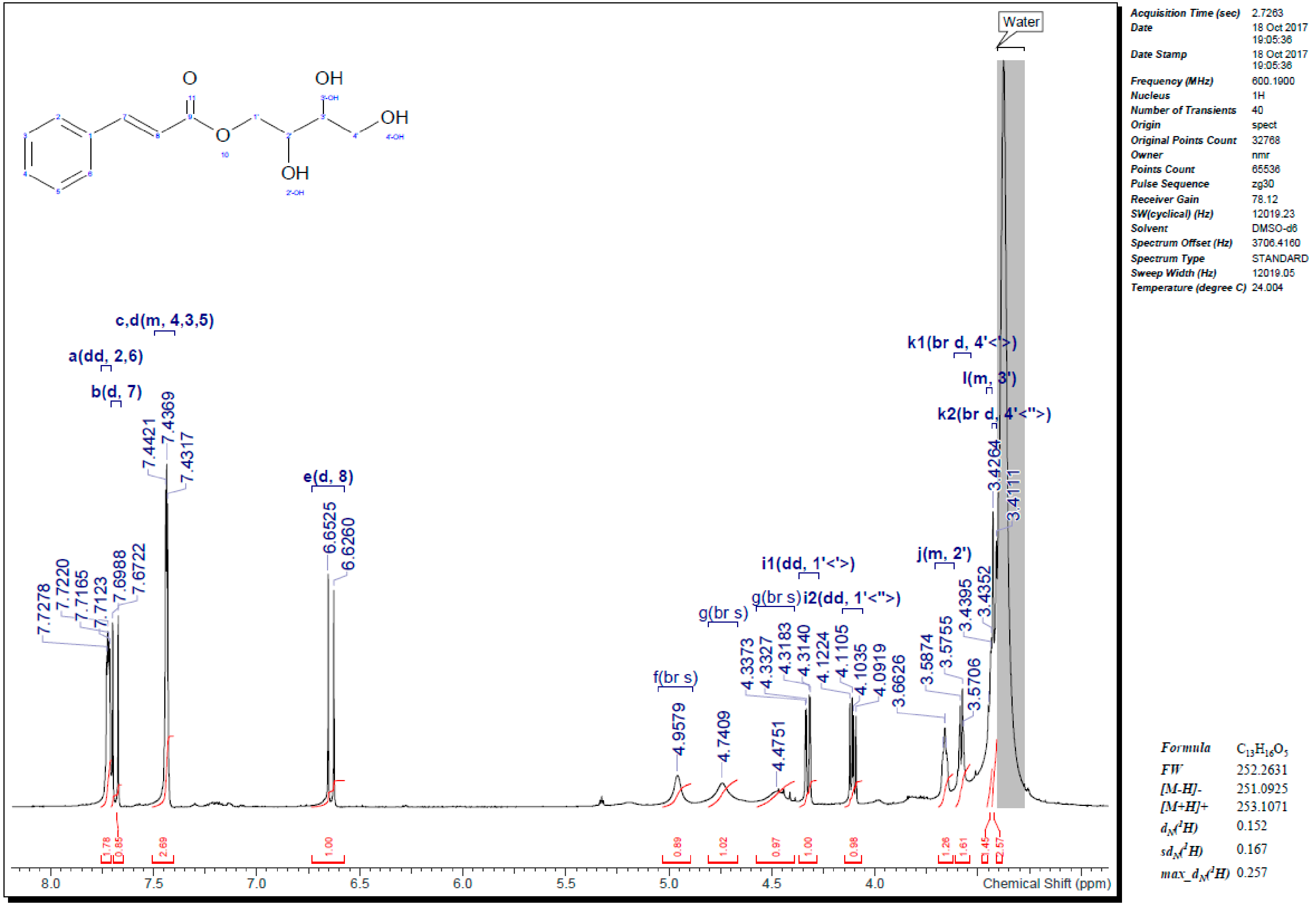

Figure S 36. ^{1}H-NMR (600 MHz, DMSO-d_6) of compound **5**

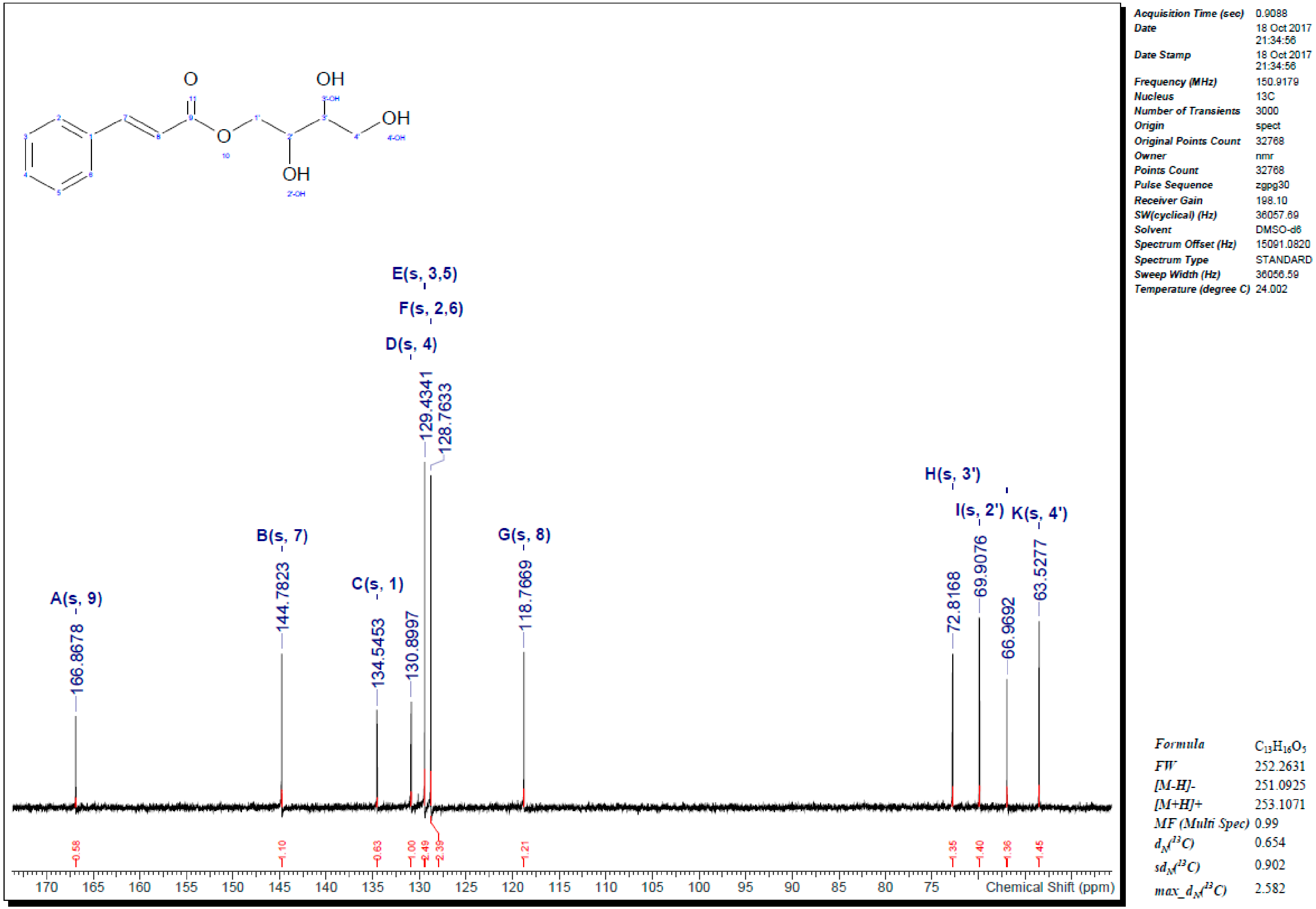

Figure S 37. ^{13}C-NMR (150 MHz, DMSO-*d*$_6$) of compound **5**

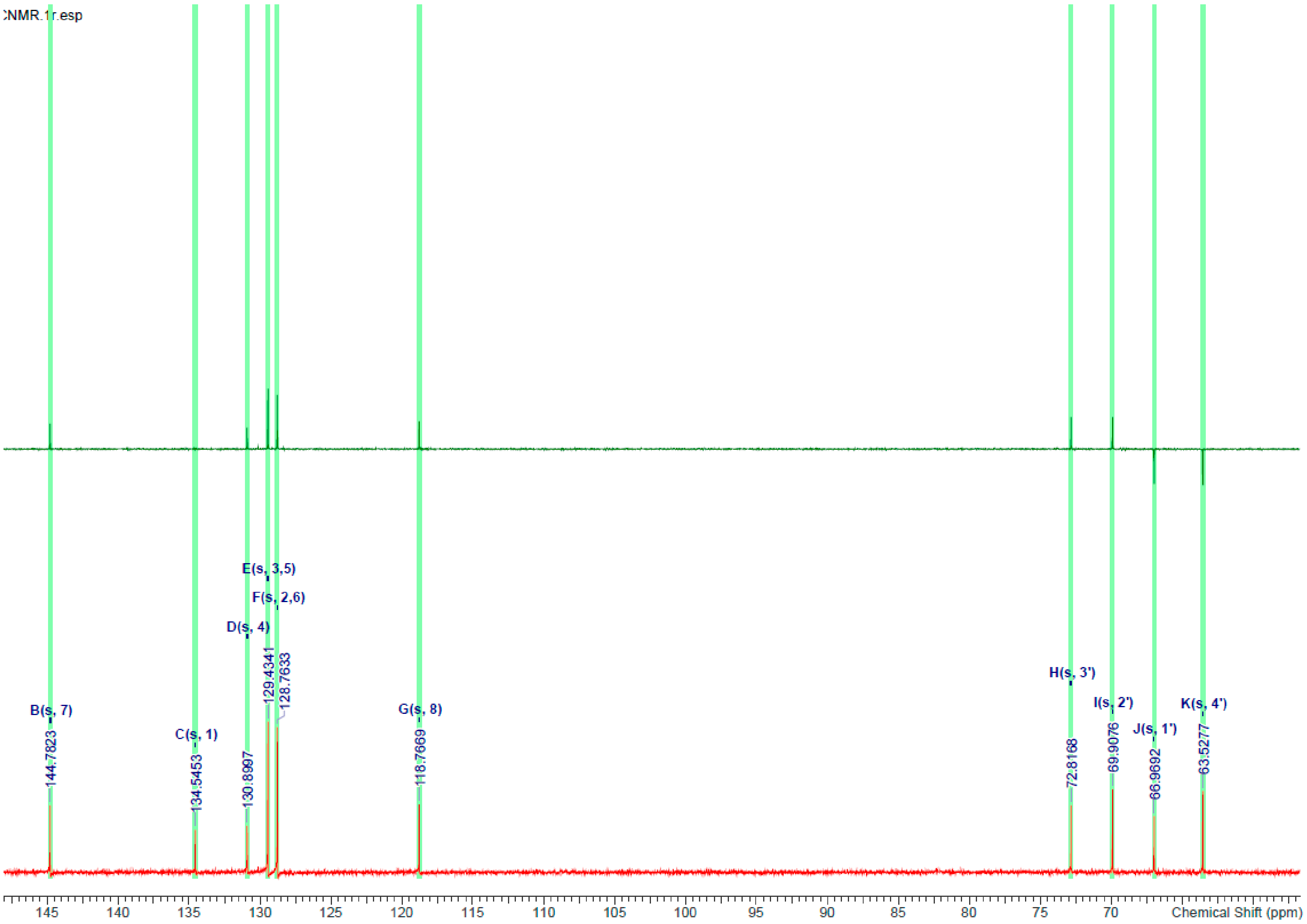

Figure S 38. DEPT of compound **5**

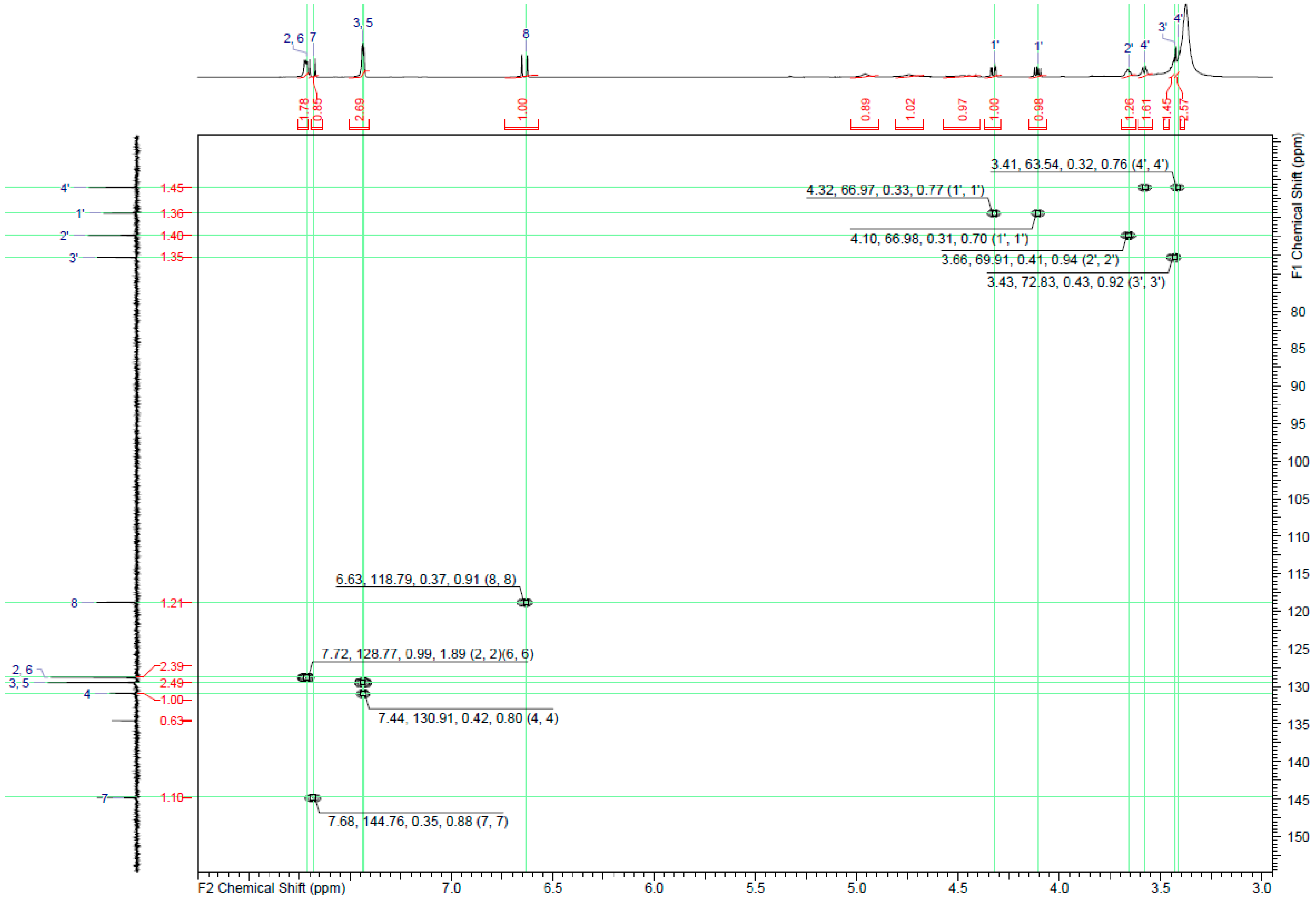

Figure S 39. HSQC of compound **5**

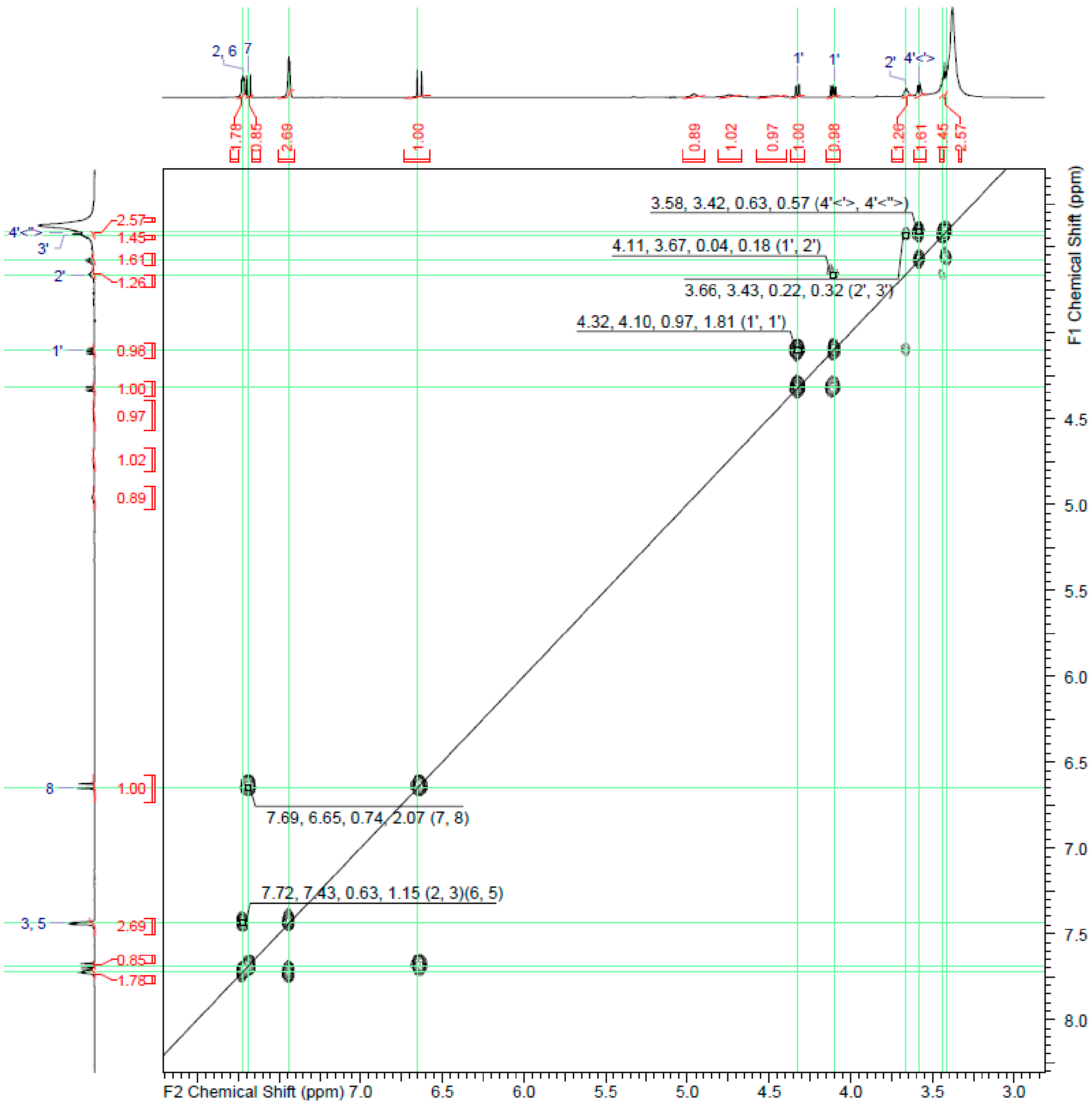

Acquisition Time (sec)	(0.0852, 0.0214)
Comment	5 mm PABBO BB/19F-1H/D Z-GRD Z114607/0133
Date	29 Oct 2017 06:06:04
File Name	C:\Users\Administrator\Desktop\yanxia-20171030\CM10-1-40%-3\3\pdata\1\2rr
Frequency (MHz)	(600.1936, 600.1936)
Nucleus	(1H, 1H)
Number of Transients	12
Origin	spect
Original Points Count	(1024, 256)
Owner	nmr
Points Count	(2048, 1024)
Pulse Sequence	cosygpmfqf
Solvent	DMSO-d6
Spectrum Type	COSY
Sweep Width (Hz)	(12013.36, 11978.70)
Temperature (degree C)	23.991
Title	

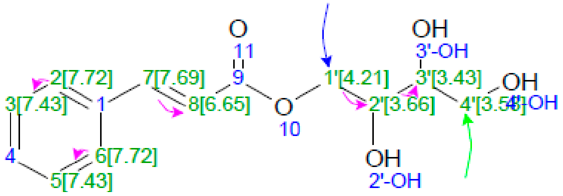

Figure S 40. ^{1}H-^{1}H COSY of compound **5**

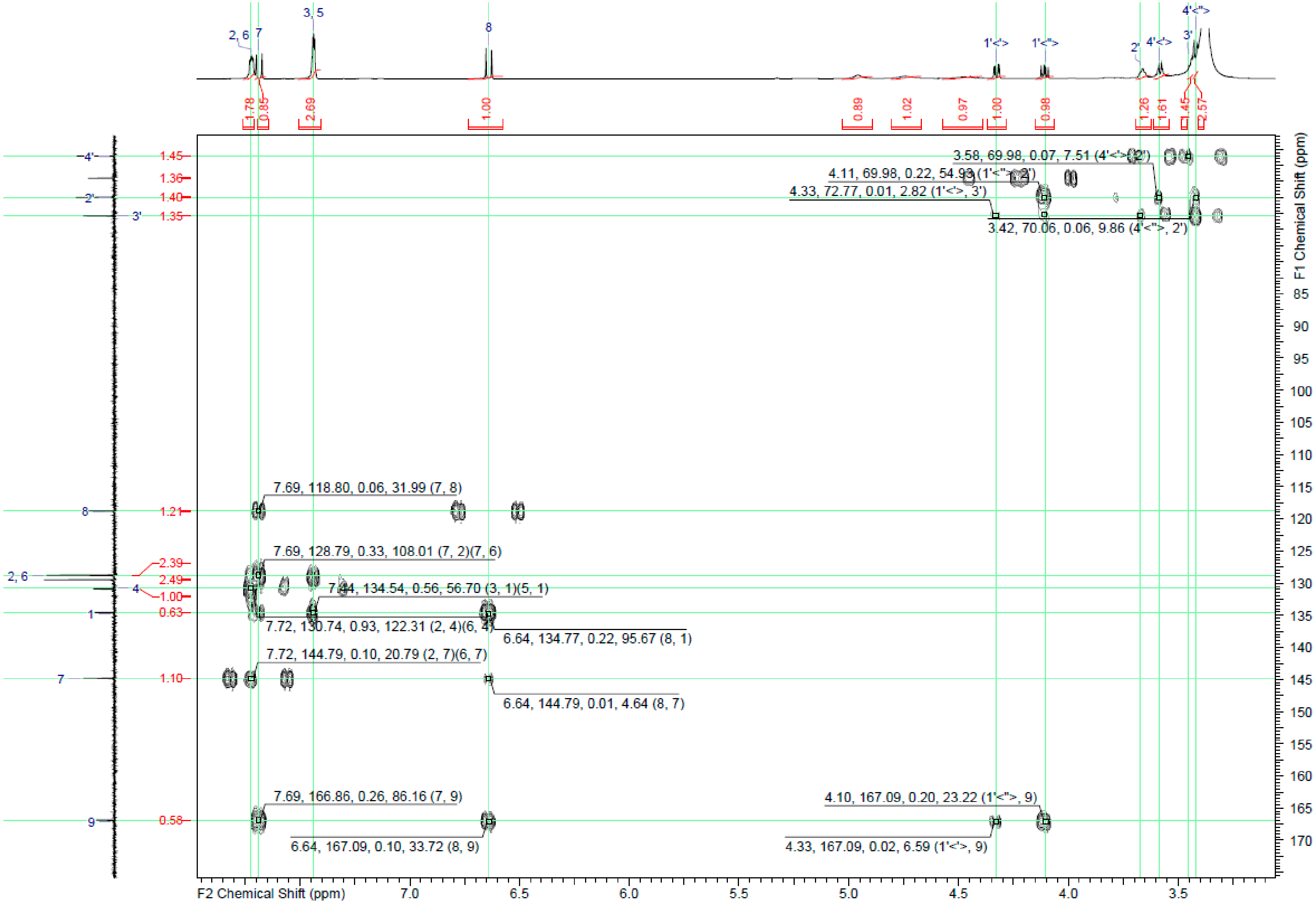

Figure S 41. HMBC of compound **5**

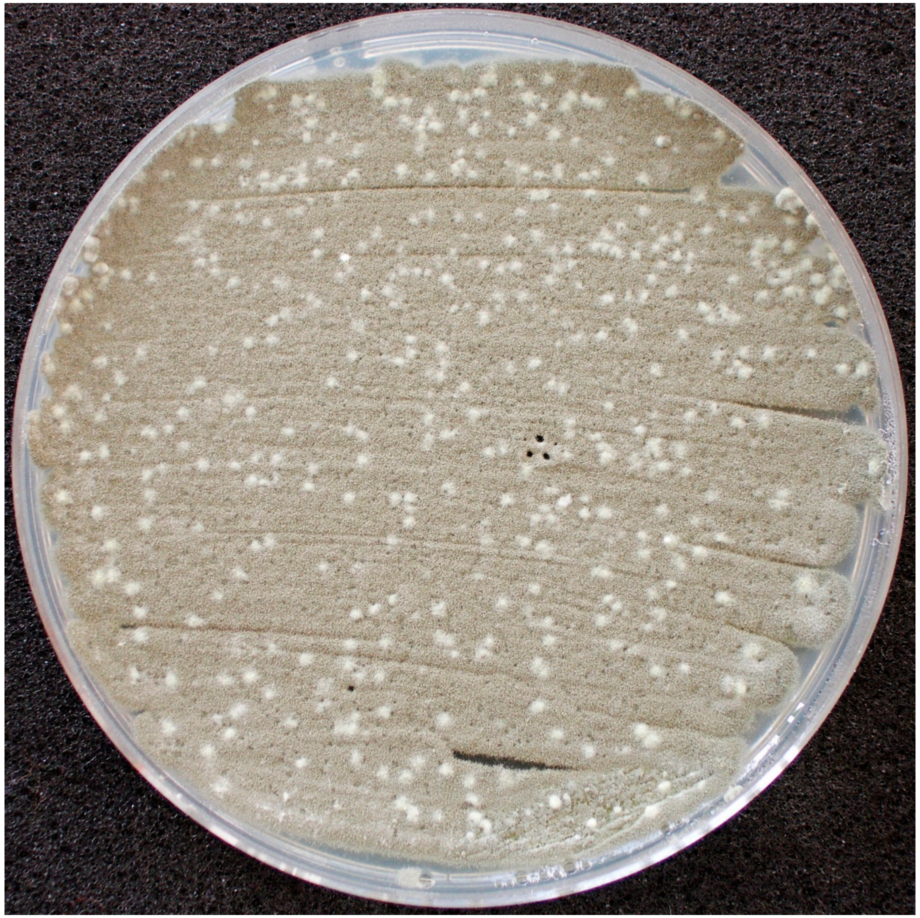

Figure S 42. Colony morphology photograph of *Penicillum citreonigrum* XT20-134.
